# Pushing the boundaries of dielectric permittivity in polysiloxanes: polar dipole modifications enable amorphous pyroelectric polymers†

**DOI:** 10.1039/d5mh00234f

**Published:** 2025-04-21

**Authors:** Patrick M. Danner, Thulasinath Raman Venkatesan, Johannes von Szczepanski, Francis Owusu, Dorina M. Opris

**Affiliations:** a Swiss Federal Laboratories for Materials Science and Technology – Empa Laboratory for Functional Polymers Ueberlandstr. 129 Duebendorf CH-8600 Switzerland dorina.opris@empa.ch; b Department of Materials, ETH Zurich Vladimir-Prelog-Weg 5 Zurich CH-8093 Switzerland

## Abstract

The main drawback of polymers in a wide range of soft electrical applications is their low dielectric permittivity. The chemical modification of polymers with organic dipoles has been a successful strategy to increase their dielectric permittivity. However, what is the maximum achievable dielectric permittivity by this method? We present four novel polysiloxanes with relative permittivities ranging from 23 to 31 at room temperature (RT), reaching 34 at 40 °C. These are the highest dielectric permittivity values reported for any amorphous filler-free elastomer. Additionally, we derive a universal guiding principle in designing future elastomers, with an ideal trade-off between elasticity and permittivity at an operating temperature of *T*_g_ + 60 °C. We further explore the resulting composites with SiO_2_ and TiO_2_ and show that two glass transitions (*T*_g_s) occur due to the interfacial layer and the bulk phase. The two phases show distinct dielectric behavior, which we demonstrate as useful in achieving pyroelectric materials. The materials exhibit the highest reported pyroelectricity of any crystal-free, fully amorphous polymer with a stable quasi-static pyroelectric coefficient of 3.4 μC m^−2^ K^−1^ at 30 ± 0.5 °C.

## Abstract 2

A polysiloxane containing nitroaniline has a permittivity of 35 at 40 °C. Its TiO_2_ composite has two *T*_g_s for the polymer in bulk and interface. Poling of this composite leads to a material with a pyroelectric coefficient of 3.4 μC m^−2^ K^−1^ at 30 °C.

## Graphical abstract

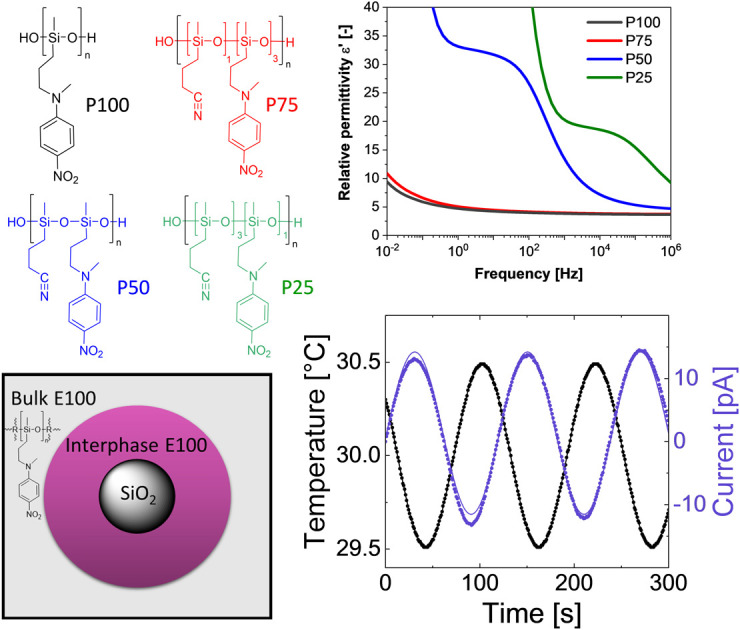

**New concepts**

Soft, high-dielectric permittivity materials are essential for actuators, sensors, energy harvesting, and capacitive light-emitting device applications. However, synthesizing polar elastomers simultaneously exhibiting high dielectric permittivity and a low glass transition temperature remains a significant challenge. Here, we demonstrate a novel molecular design strategy that modifies the highly flexible polysiloxane backbone with a synergistic combination of cyanopropyl and nitroaniline push–pull dipoles. This approach enables a record-high dielectric permittivity of up to 35 at 40 °C in a neat elastomer, representing a 12-fold increase over standard polydimethylsiloxane. What differentiates our concept from existing research is its reliance on molecular-level modifications rather than traditional filler-based enhancements. Unlike previous approaches, which often compromise mechanical flexibility, our strategy allows achieving a unique balance between elasticity and dielectric performance, paving the way for intrinsically high-permittivity elastomers. Beyond enhancing dielectric properties, our work introduces a fundamentally new concept for achieving stable pyroelectric responses in amorphous polymers through strategic nanocomposite and interface engineering. By leveraging this approach, we develop materials with a pyroelectric coefficient of 3.4 μC m^−2^ K^−1^ at 30 °C after 41 days, outperforming other reported amorphous pyroelectric materials. Our findings provide insights into high-dielectric permittivity elastomers, paving the way for next-generation dielectric and pyroelectric materials.

## 1. Introduction

Dielectric elastomers have a wide range of applications in dielectric elastomer transducers (DET), including strain, pressure and force sensing as dielectric elastomer sensors (DES), electrically driven actuation as dielectric elastomer actuators (DEAs), energy harvesting as dielectric elastomer generators (DEGs), for energy storage devices such as elastic capacitors or for electroluminescence in alternating current electroluminescent (ACEL) devices or as elastic transistors.^1–7^ These applications are plentiful and are crucial in many aspects of stretchable electronics, artificial skin, and soft robotics.^8,9^ Unfortunately, the low permittivity of elastomers is a major drawback for these applications. High-permittivity dielectric elastomers have been achieved through two approaches.^10,11^ The first is a composite approach, where the elastomer is filled with highly polarizable fillers by incorporating ceramics, conductive fillers, liquid metal, or softeners such as PEG, glycerol, or ionic liquid into a polymer matrix.^10–21^ The second approach is the chemical modification of polymers with polar side groups.^1,10,11,22–30^ This strategy yields neat high-permittivity elastomers that could be further turned into composites with different functionality. In recent years, the modification of various backbones with different polar groups has led to a better understanding of the impact of chemical modification on the properties of the resulting dielectric elastomer.^10,22^ While various polymers have been explored, polysiloxane-based elastomers are among the most promising backbones for high-permittivity elastomers due to their outstanding mechanical properties and low glass transition temperature (*T*_g_).^10,22^ The low *T*_g_ allows for achieving polar materials with room temperature elasticity. Recent work from our laboratory presented a variety of polar groups for the modification of polysiloxanes yielding high-permittivity polar polysiloxanes.^22^ The highest permittivity was reported for a polysiloxane modified with carbonate groups, reaching a permittivity of ∼28 with a *T*_g_ = −18.2 °C.^22^ Polysiloxanes modified with a cyano group at every repeating unit show a permittivity of ∼18 and a fairly low *T*_g_ = −59.8 °C.^31–34^ The cyano group has a moderate dipole moment of 3.9 D.^35^ According to the Onsager equation valid for polar nonassociating liquids, the static dielectric permittivity (*ε*_s_) can be calculated if the dipole moment (*μ*) is known.^36^
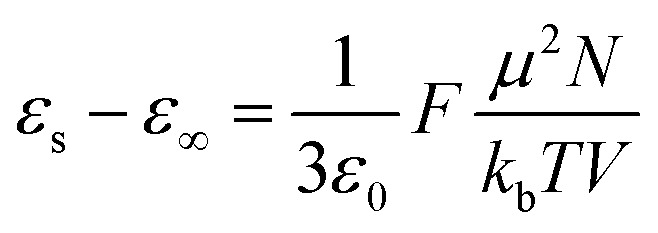
where *ε*_∞_ is the high-frequency permittivity (*ε*_∞_ = *n*^2^), *n* is the refractive index, *N*/*V* is the number of dipoles per unit volume, *T* is the temperature, *ε*_0_ is the vacuum permittivity, *k*_b_ is Bolzmann constant.
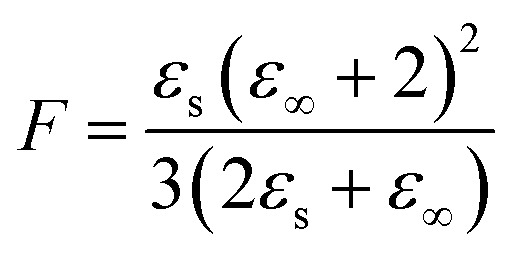


The equation shows that the dielectric permittivity should increase with the dipole strength. So, by replacing the cyano groups on this polymer with an even stronger dipole, we should achieve even higher dielectric permittivity values. Assuming the *T*_g_ is still low enough, the polar groups should still be polarisable at room temperature, contributing to the increase in permittivity *via* the dipolar orientation. A polysiloxane with about 25 mol% of the repeating units modified with nitroaniline (NA) groups exhibits a permittivity of ∼18 and a *T*_g_ = −35 °C.^37,38^ This is due to NA's high dipole moment of 6.9 D.^39^ Since 75 mol% of the repeating units in this polymer consisted of non-polar dimethylsiloxane, we wanted to elucidate how polysiloxanes modified with varying amounts of both cyanopropyl (CN) and NA polar groups will influence the dielectric permittivity and the mechanical properties of the resulting elastomers.

In addition, little attention has been paid to how different fillers affect the dielectric properties of high dielectric permittivity polar polymers with low *T*_g_. This is important as most elastomers used in applications contain some filler to tune their mechanical properties. In the past, it was found that adding metal oxide nanoparticles such as SiO_2_ and TiO_2_ into elastic materials leads to the adsorption of the polymer chains onto the metal oxide, forming an interfacial layer.^40,41^ The interfacial layer has been shown to follow retarded dynamics than the bulk material, leading to an additional *T*_g_.^40,42,43^ The addition of SiO_2_ or TiO_2_ nanoparticles in a PDMS matrix has been shown to lower the *T*_g_ of PDMS and, at the same time, lead to an additional *T*_g_ due to the adsorbed polymer chains.^41,43,44^ This interfacial *T*_g_ is observed at a higher temperature than the bulk *T*_g_ due to the restricted movement of the adsorbed chains. Much effort has been made by Kumar *et al.* and also by Pissis *et al.* towards understanding the influence of the metal oxide filler type,^41^ size,^45^ shape,^46^ curvature,^47^ surface roughness,^48^ method of preparation^49^ and the influence of polymer molecular weight^50–52^ on the interfacial adsorbed layer properties and characteristics. According to the models proposed by Klonos and co-workers, both the strength of the interphase (*α*_int_) relaxation and its relaxation time scale depend on the first polymer layer, which is adsorbed onto the metal oxide surface.^48,50,51,53^ Initially, the multiple –OH groups on the surface lead to the adsorption of the polymer at multiple points.^50,51^ This results in a so-called dead polymer segment, which is immobile. However, they can have tails or form loops, exhibiting limited mobility. Especially, in the case of polymer chains below their entanglement threshold, extended tails are formed.^51^ While the tails have bulk-like density with reduced cooperativity, the loops have a higher density and cooperativity.^48^ The extent to which these tails and loops can be present on the adsorbed layers depends on the previously mentioned parameters.

However, these investigations have been performed on low permittivity elastomers, mostly PDMS, and the observed effects have little impact on the dielectric and mechanical behavior at room temperature. Consequently, the observed interfacial effects could not be used for applications. In high-permittivity elastomers, the retarded alignment of the interfacial polymer layer, however, has consequences on the dielectric behavior even at room temperature, as we previously observed.^34,54^ This opens the possibility to use the interfacial effect for applications such as pyroelectric energy harvesting.

**Fig. 1:**
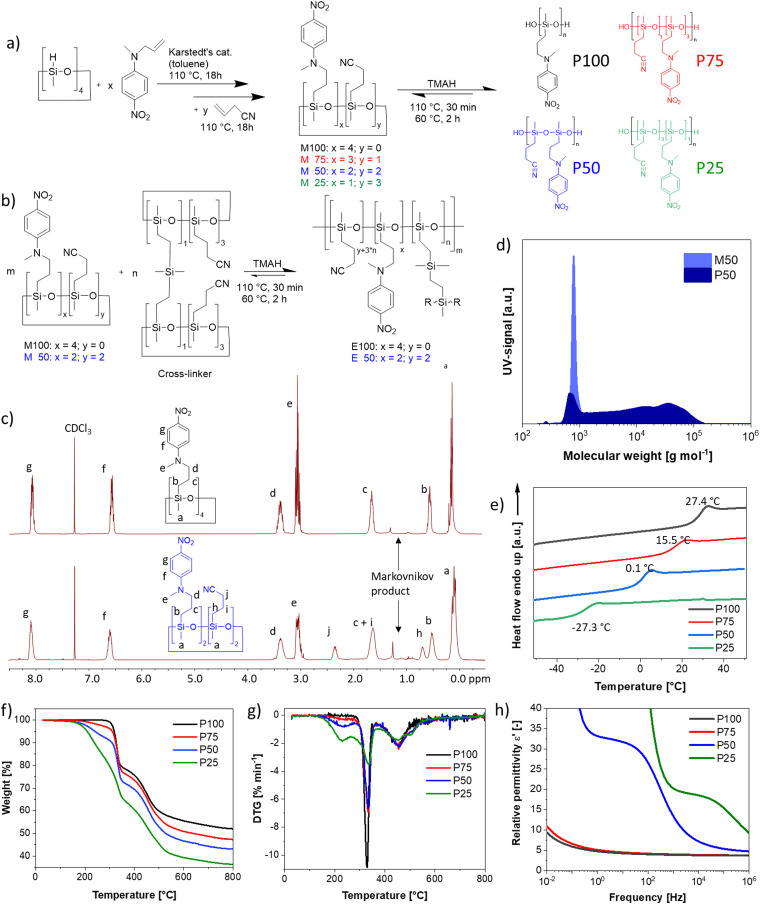
(a) One-pot synthesis of polar cyclosiloxanes modified with nitroaniline (NA) and cyanopropyl (CN) groups. First, the NA is grafted, followed by the CN groups. The content of NA and CN groups on the monomer can be varied yielding monomers M100 (100 mol% NA), M75 (75 mol% NA/25 mol% CN), M50 (50 mol% NA/50 mol%), and M25 (25 mol% NA/75 mol% CN). Anionic ring opening polymerization of monomers leads to polymers P100, P75, P50, and P25, respectively. (b) M100 and M50 are polymerized in the presence of a bifunctional cross-linker leading to the elastomers E100 and E50, respectively. (c) ^1^H NMR spectra of M100 (top), and M50 (bottom) in CDCl_3_. The letters indicate the respective proton signal and the peaks between 0.75 and 1.5 belong to the Markovnikov product of the hydrosililation reaction. (d) Gel permeation chromatography (GPC) elugrams of M50 and P50. (e) Differential scanning calorimetry (DSC) of P100, P75, P50, and P25 from −50 up to +50 °C. (f) Thermogravimetric analysis (TGA) of the four polymers from 25 up to 800 °C and (g) weight change with temperature. (h) Broadband dielectric spectroscopy performed on all four polymers at room temperature with AC frequency of 0.01 Hz up to 1 MHz.

Here, we present polysiloxane elastomers modified with different content of NA and CN groups (Fig. 1). The resulting elastomers have the highest reported relative permittivity of any neat elastomer. The permittivity is significantly higher than in PDMS and can reach values as high as 31 at RT. Achieving neat elastomers with even higher permittivity by this method seems unlikely, as we showcase that chemical modification results in significant shifts in *T*_g_, heavily impacting the mechanical properties of the elastomers. We derive a guiding principle of *T*_g_ + 60 °C as an ideal operating temperature for obtaining the highest possible permittivity coupled with decent elasticity. Further, we showcase fillers' effect on these high permittivity elastomers, leading to different properties for the bulk and interface polymer phases. Since elastomers cannot store (remanent) dipolar polarization, amorphous elastomers cannot inherently exhibit pyro- and piezoelectric properties found in several crystalline and semi-crystalline materials. Therefore, significant research has been focused on investigating how to turn elastomer piezoelectric.^55,56^ We exploit the presence of two distinct phases, despite having a homopolymer, and showcase their application for pyroelectric energy harvesting. In this scenario, we have partially polarized dipoles at the interphase and relaxed dipoles in the bulk. The material exhibits pyroelectric response within the temperature range between the two *T*_g_'s, where the change in polarization over a temperature change occurs due to the change in dipole density (secondary pyroelectricity).

## 2. Results and discussion

### 2.1. Polysiloxanes with varying CN & NA content

We developed a two-step synthesis strategy for polar polysiloxanes with varying NA and CN contents (Fig. 1a). First, *N*-allyl-*N*-methyl-4-nitroaniline is reacted with 1,3,5,7-tetramethycyclotetrasiloxane (D_4_H_4_), followed by the addition of allyl cyanide, leading to a full-conversion of hydrosilyl groups and a statistical distribution of the polar groups on the monomers. Both addition reactions are performed with an efficient hydrosilylation reaction catalyzed by Karstedt's catalyst. Tuning the amount of *N*-allyl-*N*-methyl-4-nitroaniline and allyl cyanide allowed to increase the NA content on the D_4_H_4_ monomers from one, two, three to four groups while reducing the content of CN from three, two, one, to zero, respectively. The varying content of NA and CN groups on the monomers allows for tuning the dielectric permittivity and the *T*_g_ of the resulting polymers. The monomers are named according to the NA content, where M100 refers to the monomer with 100 mol% NA, M75 refers to the monomer with 75 mol% NA and 25 mol% CN, M50 refers to the monomer with 50 mol% NA and 50 mol% CN, and M25 refers to the monomer with 25 mol% NA and 75 mol% CN (Fig. 1a). The monomers are then *in situ* polymerized and cross-linked by a multifunctional monomer, as will be explained later (Fig. 1b and Fig. S1, ESI†). The successful synthesis of the monomers M100 and M50 was proven by ^1^H NMR spectra shown in Fig. 1c and for M75 and M25 in Fig. S2 (ESI†). In addition to the aromatic signals due to the grafting of NA, the most notable change is the rise of two new ^1^H NMR signals caused by the addition of the CN groups (labeled as j and h in Fig. 1c). It is noteworthy that more Markovnikov additions occur with more CN groups incorporated. Typically, the Markovnikov addition in a hydrosilylation is below 10%. The Markovnikov addition in M100 is 7.8%, increasing by 8.1% for M75, 12.3% for M50, and 14.8% for M25. This indicates that the cyano group interferes with the efficiency of the hydrosilylation reaction. The poisoning of the Pt-catalyst by the cyano group has been repeatedly reported.^32,33,57^ Indeed, cyano groups are often used as Pt-inhibitors, as they can form stable complexes with the Pt catalyst.^58^ Nonetheless, the reaction proceeded to completion; however, it was not as fast as typical for hydrosilylation reactions. Anionic ring opening polymerization (AROP) of the monomers initiated by tetramethylammonium hydroxide (TMAH) gave polymers P100, P75, P50, and P25 (Fig. 1a). Please note that the polymers' composition follows a statistical distribution of polar groups. The only exception is homopolymer P100, which is fully functionalized with NA. The ring-opening polymerization of cyclosiloxane is an equilibrium reaction that produces a mixture of cycles and chains, typically in a 1 : 9 ratio, due to the ability of active chain ends to propagate and undergo backbiting.^59^ As long as the chain ends remain active, elevated temperatures can shift the equilibrium toward higher cyclic components. Recent studies have shown that polar cyclic siloxanes are more temperature-sensitive than their non-polar counterparts, with higher temperatures favoring backbiting and cycle formation, whereas room temperature favors polymerization.^31,33,60^ Solvents can also significantly impact the amount of cycles formed through backbiting. To lower the backbiting reaction, we performed the reaction solvent-free. Polymerization of M50 leads to 77.7% polymer P50 with a molecular weight of *M*_n_ = 10 153 g mol^−1^ (Fig. 1d) and 22.3% low molar mass product. The low mass product is higher than typically for the AROP of PDMS, and tuning the reaction conditions is required. The typical polymerization temperature of 110 °C led to about 27.0% of low-mass products and a lower *M*_n_ = 8837 g mol^−1^, while deactivation of TMAH by heating to 150 °C for 30 minutes led to an increase of the low-mass products to about 38.7% (Fig. S3, ESI†) and an even lower *M*_n_ = 7719 g mol^−1^. Consequently, instead of thermally deactivating the active chain ends, we washed the polymer three times with water. The reaction was conducted at 110 °C for 30 minutes, followed by shifting the equilibrium towards the polymer by heating at 60 °C for two hours.

The initial heating to 110 °C is needed to allow a homogenous mixing of TMAH, as no solvent was used to reduce the backbiting, the polymerization mixture is highly viscous for all four monomers at RT and even at 60 °C. Irrespective of the workup, the polymers have a wide polydispersity index (*Đ*) of 2.8, slightly higher than those obtained in a typical AROP (around 1.8–2.2). The polymers were not further purified before characterization, as the siloxane equilibration is employed again in the *in situ* cross-linking strategy to elastomers (Fig. 1b). Consequently, the cyclic components would be formed again during polymerization/cross-linking reactions. The polymers' thermal transitions were investigated by DSC (Fig. 1e). All four polymers show only one *T*_g_ and no crystallization. The fact that only one *T*_g_ is visible indicates that a random copolymer has been obtained and that the cyclic components are blended into the polymers. Polar elastomers with only CN or NA groups have shown no crystallizations. Therefore, it is not surprising that polymers containing both CN and NA groups also do not crystallize.^34,38,61^ According to DSC, the polymers have a *T*_g_ of 27.4 °C, 15.5 °C, 0.1 °C, and −27.3 °C for the polymers P100, P75, P50 and P25, respectively. This is in good agreement with previously published polymers, with 25 mol% of the repeating units modified with NA and 75 mol% of the repeating units being dimethylsiloxane with a *T*_g_ of −38 °C and the one that carries at every siloxy repeating unit, a CN group with a *T*_g_ of −59 °C (Fig. S4, ESI†).^31,61^

According to thermogravimetric analysis (TGA), all polymers are stable up to temperatures of 150 °C (Fig. 1f). There is a distinct difference between the CN-containing polymers P75, P50, and P25 and the only NA-containing polymer P100, which is stable up to 305 °C. There are three distinct decomposition steps. First, the CN group is decomposed. The P75 loses 2.5 wt% until 270 °C, in accordance with the calculated total weight of the CN group, which is 2.9 wt%. P50 loses 7.1 wt%, with the calculated amount of 50 mol% CN groups is 6.8 wt%. P25 loses 15.3 wt% in this initial decomposition step of the CN group, where 12.3 wt% corresponds to the calculated weight loss of the CN group. In the next step, the NA is decomposed, which occurs exactly at the same temperature range as reported previously for NA-containing polysiloxanes.^61^ In the final distinct stage, polysiloxane degrades at temperatures reaching 540 °C. The decomposition of the CN group begins at 160 °C, peaks at 227 °C, and completes at 270 °C (Fig. 1g). The NA group starts decomposing at 290 °C, peaks at 330 °C, and finishes at 350 °C. Polysiloxane degradation begins at 380 °C, peaks at 458 °C, and finishes at 540 °C.

The polymers were further analyzed by impedance spectroscopy (Fig. 1h). P25 reaches a permittivity of 20.0 at 1 kHz, while P50 reaches about 33 at 1 Hz. This is the highest reported permittivity of any neat polymer. The permittivity values for P75 and P100 remain low over the whole frequency range, as the *T*_g_'s are too close to room temperature, leading to a mostly frozen-in system in both cases. The permittivity values of the polymers at RT depend on their respective *T*_g_. Therefore, we performed a more in-depth analysis of the permittivity and its change with temperature in Section 2.2.

### 2.2 Dielectric analysis of high-permittivity polysiloxanes

**Fig. 2:**
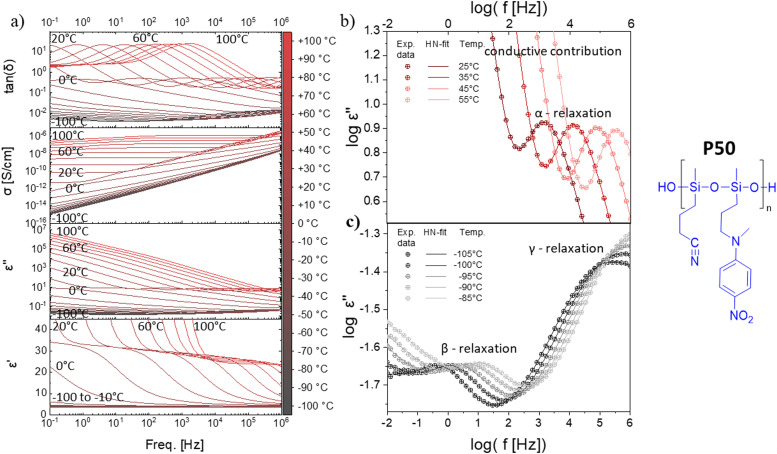
Dielectric relaxation spectroscopy of polymer P50. (a) permittivity (*ε'*), imaginary part of the permittivity (*ε*′′), ionic conductivity (*σ*), and loss factor (tan(*δ*)) at varying frequencies and temperatures. (b) Experimental data of log *ε*′′ plotted as a function of the frequency and the HN-fit function at different temperatures above *T*_g_. (c) Experimental data of log *ε*′′ plotted as a function of the frequency, as well as the HN-fit function at different temperatures below *T*_g_.

All four polymers show dielectric behavior with strong dielectric permittivity variations with temperature and frequency. The data is shown here for polymer P50 as an example, but the analysis has been performed on all four polymers, and the dielectric data can be seen in Fig. S5–S7 (ESI†). The polymer P50 behaves differently above and below the *T*_g_, as reflected by the dielectric permittivity values (Fig. 2a). At temperatures above 0 °C, the permittivity increases with decreasing frequency from around 3.2 to a plateau at ∼32 due to the orientation of the dipoles, before increasing further due to the conductive contribution and electrode polarization (Fig. 2a). At temperatures below 0 °C, the permittivity stays approx. constant over all frequencies at 3.2, as the polymer backbone has no segmental motion. Hence, the dipoles are effectively frozen in their place and cannot align along the electric field. The conductivity strongly depends on temperature and frequency and varies from 10^−14^ S cm^−1^ (−100 °C) to 10^−7^ S cm^−1^ (+100 °C). At 20 °C, the conductivity is low with ∼10^−10^ S cm^−1^ (100 Hz AC and below). The loss factor tan(*δ*) indicates three relaxation regimes, one above and two below the *T*_g_. All relaxation processes can be better observed in the imaginary part of the permittivity (*ε*′′) (Fig. 2b and c). In Fig. 2b, at temperatures above the *T*_g_, the α-relaxation and the electrode polarization due to the conductive contribution can be observed and the Havriliak–Negami (HN) relaxation function was fitted through the data points. The processes below the *T*_g_, namely the β- and γ-relaxation, and their respective fitted HN-function can be observed in Fig. 2c. The data show two distinct relaxation processes below the *T*_g_, following the classical theoretical description of glass-forming polymers.^62^

**Fig. 3:**
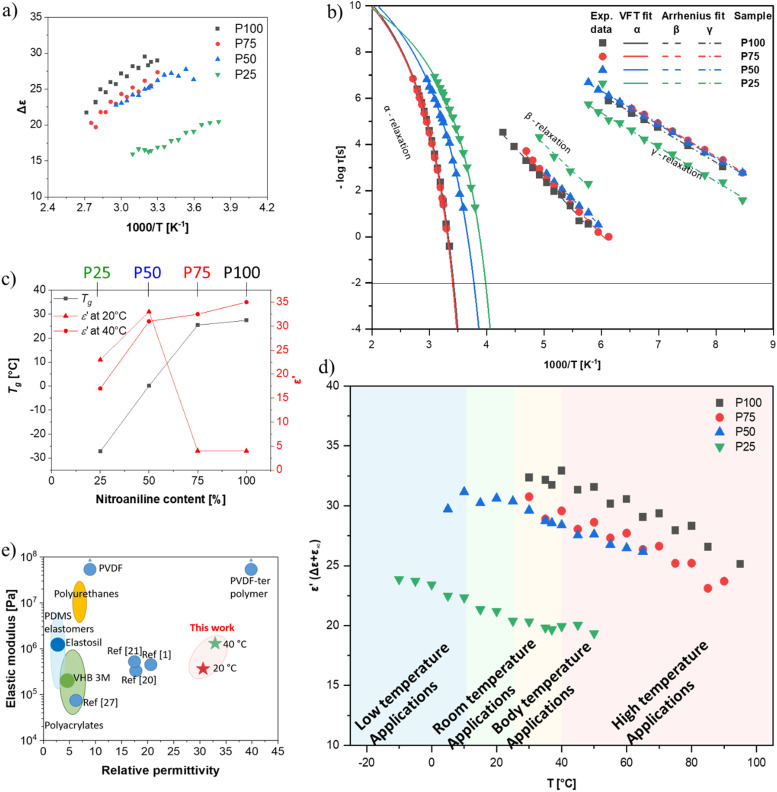
(a) Relaxation strength (Δ*ε*) of the dipoles extracted from the HN-fit at different temperatures (b) Arrhenius plot of the four polymers with VFT-fit for the α-relaxation and Arrhenius-fits for the β and γ (c) dependency of *T*_g_ and permittivity with varying nitroaniline and cyanopropyl content and temperature. (d) Absolute permittivity of the different polymers at different temperatures extracted from the relaxation strength (Δ*ε*) and background permittivity (*ε*_∞_; values taken at −150 °C and 10^6^ Hz). (e) Comparison of the relative permittivity and material elastic modulus with materials from literature.

The HN-fit gives the relaxation strength (Δ*ε*) for all four polymers (Fig. 3a). Polymer P100 reaches a relaxation strength of Δ*ε* = 30 at 40 °C. To our knowledge, this is the highest dipole relaxation strength of a polysiloxane ever reported.^63,64^ The other three synthesized polymers show lower values but are still among the highest reported in polysiloxanes. They follow a clear trend, where the relaxation strength increases with increased NA concentration. Additionally, with a continuous increase in temperature beyond the *T*_g_, the relaxation strength decreases, which can be explained by the thermal expansion of the polymer, leading to dipole density dilution and the high entropic energy of the dipoles at high temperatures, which limits their polarizability. At lower temperatures, however, the dipole relaxation strength does not gradually decrease but drops suddenly. This is due to the freezing of the chain movement, which means the dipoles can no longer orient within the electric field. Hence, the materials have a low permittivity below their respective *T*_g_, with only low contributions from β- and γ-relaxations and electronic and atomic polarization.

Apart from the relaxation strength, the HN-function also gives the relaxation times of each polymer at varying temperatures, as can be seen in Fig. 3b. The fastest observable process was the γ-relaxation. The obtained data follows the Arrhenius fit, from which the activation energy could be extracted to be approx. 52 kJ mol^−1^ for all polymers. The β-relaxation is slower than the γ-relaxation, while the Arrhenius-fit has an activation energy of around 27 kJ mol^−1^. Lastly, the α-relaxation follows the Vogel–Fulcher–Tammann (VFT) fit, from which the *T*_g_ can be extrapolated for all four polymers. According to VFT, the P100, P75, P50, and P25 polymers have a *T*_g_ of 27 °C, 22 °C, 0 °C, and −26 °C, respectively, which is in good accordance with the *T*_g_ values obtained from DSC analysis (Fig. 1e). As previously discussed, the *T*_g_ decreases with increased CN content in our polymers (Fig. 3c). Consequently, the permittivity of our polymers is highly temperature-dependent. The polymers with concentrations of up to 50% NA can be used at room temperature, while the polymers with higher NA content can only be used for body-temperature or high-temperature applications (Fig. 3d). Thus, the four polymers can be used for different application fields, exhibiting remarkably high permittivity. Polymer P100 has a dielectric permittivity of 34 at 40 °C (Fig. 3d), which may be interesting for applications within or near the body. Additionally, the strong switch of permittivity at 20–40 °C allows this material to be used in temperature sensing or pyroelectric energy harvesting, as we describe below. Polymer P50 is an interesting candidate for dielectric applications at room temperature. Fig. 3e shows that the elastomers made from these polymers have the highest reported permittivity of amorphous elastomers combined with typical elastic moduli of PDMS.

### 2.3. Elastomers with varying NA & CN content

From the previously discussed polymers, the most interesting ones are P50 (*ε*′ = 31 at 100 Hz, RT) for RT applications and P100 (*ε*′ = 34 at 100 Hz and 40 °C) for body applications. Both polymers were successfully cross-linked with 10 wt% polar cross-linker (Fig. 1b and Fig. S1, ESI†) to give elastomers E100 and E50. The cross-linking is achieved through a second polar multifunctional monomer (Fig. 1b), previously reported.^33^ For the cross-linker synthesis, D_4_H_4_ was first reacted with dimethyldivinylsilane to chemically bridge two or more tetracyclosiloxane monomers, followed by the conversion of the unreacted hydrosilyl groups with allyl cyanide. The matching polarity of the cross-linker and M100 and M50 monomers is advantageous, as previous work showed that a difference in the polarity of monomers leads to phase separation.^34^ This rather simple synthetic strategy allows for tuning the content of NA and CN dipoles and for a random cross-linking, forming a dynamic network that can be reprocessed, as described in our earlier work.^60^ The cross-linked elastomers have a high permittivity and a modulus between 0.5 MPa and 3 MPa, typical ranges for polydimethylsiloxane elastomers.

**Fig. 4:**
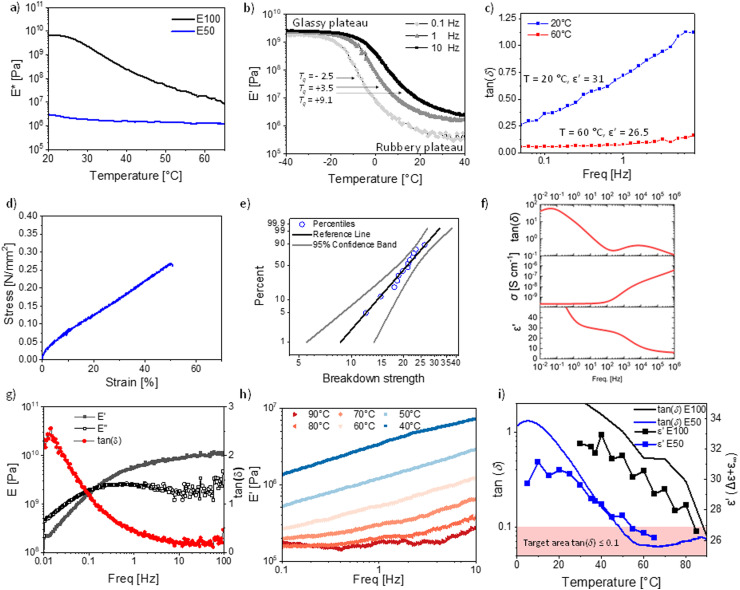
The two most promising materials E100 and E50, were analyzed by: (a) dynamic mechanical analysis (DMA) from 20 to 70 °C at 10 Hz and 0.1% strain. (b) E50 temperature sweep at 0.1% strain from 0.1, 1 to 10 Hz from −40 to +40 °C. (c) E50 frequency sweep loss tangent at 1% strain at 20 and 60 °C. (d) E50 stress–strain curve at RT. (e) E50 Weibull plot for the electrical breakdown probability at RT. (f) E50 dielectric spectroscopy data of the cross-linked material at room temperature. (g) E100 frequency sweep from 0.01 to 100 Hz at 0.1% strain and 20 °C. (h). E100 frequency sweep at 1% strain from 0.1 to 10 Hz and the *E*-modulus at temperatures from 40 to 90 °C in increments of 10 °C. (i) E50 and E100 development of the tan(*δ*) and permittivity with temperature.

The elastomer E50 complex *E*-modulus drops from 3.01 MPa at 20 °C to a typical modulus for unfilled polysiloxanes of 1.30 MPa at 60 °C. However, the complex modulus of E100 drops from 6.6 GPa at 20 °C to 18.8 MPa at 60 °C and does not yet fully reach the rubbery plateau (Fig. 4a).

This highlights the strong difference in mechanical properties due to the elastomer *T*_g_'s of −2.5 °C for E50 (according to DMA at 0.1 Hz Fig. 4b) and 25.4 °C for P100 (according to DMA at 0.1 Hz, Fig. S8, ESI†).

The DMA of the temperature sweep from −40 to +40 °C was performed for E50 at 0.1 Hz, 1 Hz, and 10 Hz (Fig. 4b). At −40 °C, the storage modulus *E*′ is in the range of 2–3 GPa, which is typical for glassy polymers. The modulus remains constant in the glassy plateau until it drops at −20 °C (0.1 Hz), −10 °C (1 Hz), and −5 °C (10 Hz). The *T*_g_ (defined as the peak of the loss tan(*δ*)) is frequency dependent and increases from −2.5 °C (0.1 Hz), to 3.5 °C (1 Hz), to 9.1 °C (10 Hz) (Fig. 4b and Fig. S9, ESI†). These *T*_g_ values align with the values from the DSC measurement of 0.1 °C for P50 (Fig. 1e) and the VFT-fit value of 0 °C (Fig. 3b). The strong frequency dependence of *T*_g_ also affects the temperature at which the rubbery plateau is achieved. In general, the rubbery plateau is reached approx. 40 °C after the onset from the glassy plateau or +20 °C after the *T*_g_ (Fig. 4b). The rubbery plateau is reached for 0.1 Hz at 20.9 °C, 1 Hz at 26.3 °C, and 10 Hz at 36.2 °C. The moduli drop in this 40 °C temperature range (glassy to rubbery plateau) by three orders of magnitude from 2–3 GPa down to 0.36 MPa (0.1 Hz, 20 °C), 0.84 MPa (1 Hz, 20 °C) and 2.33 MPa (10 Hz, 20 °C), reaching typical moduli of polysiloxane based elastomers.

However, the *T*_g_ being so close to room temperature leads to a strong frequency dependency of the storage modulus at 20 °C, especially the loss modulus and the resulting loss tan(*δ*) (Fig. 4c and Fig. S10, ESI†). At 20 °C, the loss tangent reaches from 0.26 to 1.12 within a frequency range of 0.05 to 8 Hz. This is unusual for polysiloxane elastomers as PDMS elastomers have a *T*_g_ typically around −127 °C, resulting in only small variations with frequency at RT. To avoid this strong frequency dependency, the operating temperature of the final device needs to be even further away from the *T*_g_ of the elastomer, as exemplified by the measurement at 60 °C (Fig. 4c). At 60 °C, the losses are low with a tan(*δ*) of 0.05 at 0.05 Hz and remain low at 2.5 Hz with a tan(*δ*) of 0.10 (Fig. 4c).

Consequently, if excellent elasticity for a wide frequency window is required, such as for high-frequency (>1 Hz) DEA operations, the *T*_g_ needs to be approx. 60 °C below the operating temperature due to mechanical constraints, although such a large temperature difference is not needed for the dipole alignment in the electric field, where the highest permittivity typically reaches around 10 °C after the *T*_g_. Essentially, this lowering of the *T*_g_, to improve the mechanical properties will come at a cost of lowering the relative permittivity below the maximum levels that are reached in this work.

Nonetheless, the presented materials can be interesting for low-frequency operations in DEA, as the high permittivity leads to actuation at low electric fields with a 6% lateral actuation at 10 V μm^−1^ (Fig. S11, ESI†). However, its cyclic speed is limited, similar to the behavior of acrylic materials such as VHB with a very similar *T*_g_ of 0.13 °C.^65^ The tensile test showed a Young's modulus of 0.86 MPa, well in line with the low-frequency *E*′ modulus (Fig. 4d). The strain at break is low, with 50% strain at break. This might be another effect of the *T*_g_ being so close to RT, which affects the elasticity. However, highly polar polysiloxanes have repeatedly been shown to have low strain at breaks, even if the *T*_g_s are much lower.^1,23,32,33^ We also performed a cyclic stress–strain measurement, revealing no significant hysteresis between the cycles. Due to the low speed of 50 mm min^−1^, the deformation was elastic (Fig. S12, ESI†). The electrical breakdown strength of E50 is 22.6 V μm^−1^ with a Weibull form factor of 4.80 (Fig. 4e). This is in excellent agreement with the predicted electrical breakdown field for homogenous elastomers described by Stark and Garton, with a predicted value of 23.3 V μm^−1^.^66^ The dielectric spectroscopy revealed that the cross-linking did change the dielectric permittivity, where at 10 Hz, the permittivity of E50 is about 30 at RT, compared to the uncross-linked polymer P50 with a dielectric permittivity of 31.6 at 10 Hz (Fig. 1f). The lower permittivity is due to the slightly reduced concentration of NA, as 10 wt% cross-linker was used in the synthesis. The cross-linker reduces the content of the push–pull NA dipole. Therefore, despite the polar cross-linker, the dielectric permittivity still decreases with the amount of cross-linker used since the CN group has a smaller dipole moment than the NA.

We also analyzed the mechanical properties of the material E100 cross-linked with the same polar cross-linker. Again, E100 is highly frequency dependent at 20 °C (Fig. 4g), the material modulus changes by two orders of magnitude from frequencies between 0.01 to 100 Hz with a modulus of 0.20 GPa (0.01 Hz) up to 10.19 GPa (100 Hz). Additionally, the damping factor changes significantly from 2.56 at 0.01 Hz to 0.15 at 38 Hz. This unique behavior is due to the glass transition zone. In fact, the damping factor and *E*-modulus of the material at room temperature exceed the Ashby limit for the mechanical damping and stiffness trade-off at all frequencies. This is unique, as usually only composites exceed this limit. Recently, a high-damping factor elastomer based on a fluorinated polymer for soft robotics using this glass-transition zone has been reported.^67^E100 offers a per- and poly-fluoroalkyl substance (PFAS) free alternative with similar damping properties. We further performed a frequency sweep at temperatures from 30 to 90 °C (Fig. 4h). The modulus at 40 °C increases from 1.33 MPa (0.1 Hz) to 7.14 (10 Hz). At 60 °C, the modulus increases from 0.24 MPa (0.1 Hz) to 1.25 MPa (10 Hz). At 90 °C, the modulus is 0.21 MPa (0.1 Hz), which only increases by Δ*E*′ = 0.15 MPa to 0.36 MPa (10 Hz). As for materials E50, material E100 shows a strong frequency dependence close to the *T*_g_, while at 90 °C, which equals approx. 60 °C above the *T*_g_, the frequency dependency is significantly reduced. These results show that the above-described temperature range of a *T*_g_ of 60 °C below operating temperature is an important design parameter for high-permittivity elastomers with excellent elasticity. This can again be seen by comparing the loss factors of materials E50 and E100 (Fig. 4i). While the permittivity of the E100 elastomer at 40 °C, just above *T*_g_, is 32.9 (Fig. 4i), it drops to 25.6 at 90 °C (60 °C above *T*_g_). Analog, the E50 elastomer has a permittivity of 31.2 at 10 °C, just above *T*_g_, but a relative permittivity of 26.5 (60 °C above *T*_g_). Interestingly, both elastomers E50 and E100 achieve decent elasticity at 60 °C above their respective *T*_g_s and a very similar permittivity. We believe that the *T*_g_ + 60 °C is a universal design parameter for elastomers aiming at maximizing permittivity while achieving decent elasticity (defined as tan(*δ*) ≤ 0.1). At this temperature, the thermal expansion allows the elastomer to be flexible enough to respond to fast mechanical stimuli. At the same time, the dilution of the diploes is kept at the lowest possible level. To see if this design rule holds for other polymers, we investigated PDMS and ten previously reported functionalized polysiloxanes (Fig. S13–S15, ESI†).^22,34^ We can see that all of these polymers' permittivity's drops above the *T*_g_'s as is the case for P100 and P50. This is also the case for PDMS (Fig. S13, ESI†), which shows a permittivity of 3.1 at −70 °C (*T*_g_ + 60 °C) while it shows a permittivity of 2.7 (14% lower) at RT and 2.5 (20% lower) at 100 °C. Indeed, all previously tested polymers follow the same trend and show that the relative permittivity drops after the peak *T*_g_ + 60 °C. Unfortunately, not many temperature-dependent DMA measurements of high-permittivity elastomers are available to understand if the elasticity is indeed always at this temperature in the desired range. However, some evidence for other elastomers exist. Work by Sheima *et al.* on a polar polysiloxane with sulfonyl side groups with a *T*_g_ of −13.5 °C shows good elasticity with a tan (*δ*) < 0.1 reached at a temperature of 45 °C.^68^ This falls exactly into our predicted temperature range of *T*_g_ + 60 °C with a *T*_g_ + 58.5 °C. They also showcased that the actuation of the DEA from this material improved significantly by increasing the temperature from 20 to 40 °C. In another work, an amide functionalized polysiloxane with a *T*_g_ of −41 °C showed at RT a good elasticity with a tan(*δ*) < 0.1, again falling into our described temperature target of *T*_g_ + 60 °C.^1^ Interestingly, other types of elastomers, such as polyacrylates, also follow this proposed rule. VHB4905, with *T*_g_ of 0.3 °C, is well known for its high viscous losses at RT and exhibits a much-improved elasticity at 60 °C.^69,70^ Therefore, lowering the *T*_g_ of polyacrylates to −40 °C leads to lowering the losses at 20 °C, and lowering the *T*_g_ to −30 °C leads to low losses at +30 °C, all in line with our proposed temperature guideline.^70,71^ This indicates that the proposed *T*_g_ + 60 °C guideline can help design all types of novel elastomers that aim at optimizing permittivity while achieving decent elasticity. Consequently, for excellent RT elasticity, a *T*_g_ around −40 °C should be targeted, leading to a maximum expected relative permittivity of 25–26 for well-rounded materials. This results in a maximum relative permittivity for excellent elastic elastomers of 9.6 times higher relative permittivity compared to PDMS without sacrificing most of its excellent mechanical properties. While we are convinced that the *T*_g_ + 60 °C target is a useful guideline for creating new elastomers, we want to emphasize that this observation should be meant to guide and help new materials design and not be understood as a physical rule. There will be exceptions, and exact mechanical tan(*δ*) values do not only depend on the *T*_g_ and temperature but also the measurement frequency, the speed of the temperature ramp, and other measurement parameters, as well as the cross-linking density, the incorporated fillers, softeners, and additives.

However, the underlying physical phenomena of thermal expansion leading to a trade-off between a decrease in permittivity and an increase in elasticity should be true for most elastomers.

### 2.4. SiO_2_ and TiO_2_ composites in E100 and the composites pyroelectricity

We recently showed that SiO_2_ composites in a CN functionalized polysiloxane elastomer exhibit two different *T*_g_s due to different mobility of the polar groups present in bulk and at the filler interphase.^34,54^

**Fig. 5:**
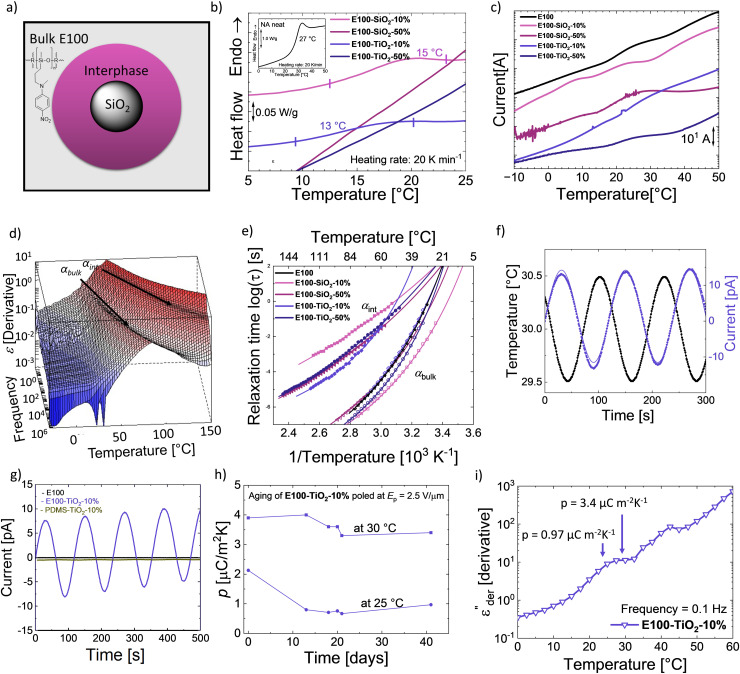
Composites of E100. (a) Illustration of the two distinct E100 phases, the bulk and interphase around the filler. (b) DSC of the different E100-MO_2_-*x*% composites and the E100 (figure inset). (c) TSDCs of E100 and E100-MO_2_-*x*% composites. (d) 3D 
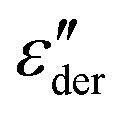
 plot of E100-TiO_2_-50%. (e) Arrhenius plot of E100 and E100-MO_2_-*x*% composites. (f) Dc conduction-free 
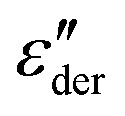
 plot as a function of temperature at 100 Hz of E100 and E100-MO_2_-*x*% composites. (g) Pyroelectricity measured on E100, PDMS-TiO_2_-10%, and E100-TiO_2_-10%. (h) Pyroelectricity measured on E100-TiO_2_-10% poled previously under an electric field (*E*_p_) of 2.5 V μm^−1^: Quasi-static pyroelectric response at 30 °C. (i) and stability of the measured pyroelectric coefficient at 25 °C and 30 °C.

The possibility of splitting the *T*_g_ of polar polysiloxane into a bulk and an interphase *T*_g_ (Fig. 5a) opens new opportunities to design novel functional materials. We exploit the rise of the second *T*_g_ due to the interfacial layer to turn composites from elastomer E100 pyroelectric. The temperature difference between the two *T*_g_s defines the operating range with broader differences, leading to a wider potential for application for such materials. Consequently, a wide difference in *T*_g_s is sought after, as well as the bulk *T*_g_ below and interphase *T*_g_ above RT. Therefore, we prepared nanocomposites with different concentrations of SiO_2_ and TiO_2_ fillers (10 and 50 wt%) in E100, resulting in composites E100-MO_2_-*x*%, where M is Si or Ti and *x* is the wt% of filler. The impact of the different fillers on the dielectric properties was investigated. In addition, their pyroelectric response was also characterized.

DSC of the different samples was measured to identify the samples' phase transitions, plotted in Fig. 5b. The neat polar polymer (figure inset) gives rise to a *T*_g_ step with a calculated value of 27 °C, agreeing with the dielectric relaxation spectroscopy (DRS) measurements (Fig. 3b). Adding the two fillers makes the transition weaker and broader in the composites, especially for the 50 wt% loaded composites, where the *T*_g_ step cannot be observed. This indicates a change in the bulk behavior of the composites and the presence of an interfacial layer. Because glass transitions are broad, two close transitions can merge into a single broad transition, and DSC is not sensitive enough to distinguish between bulk and interphase glass transitions.^40^ To better understand the phenomena, we used thermally stimulated depolarization currents (TSDCs) and DRS to complement the results from DSC (Fig. 5c, d, e and Table 1).^72^ All *T*_g_ of the nanocomposites from different techniques are tabulated in Table 1. While DSC shows only a single transition, TSDC and DRS reveal two distinct transitions in the nanocomposites. Fig. 5c shows the TSDCs measured on the various samples. The glass transition of the polar polymer in the form of a shoulder roughly between 15 °C and 30 °C can be identified.

**Table 1:** Glass-transition temperatures measured using DSC, DRS, and TSDC for E100 and different composites E100-MO_2_-*x*%

Sample	DSC		TSDC		DRS		*E*_p_	*T*_py_	*p*
	*T*_g_ [°C]	*T*_g,bulk_ [°C]	*T*_g,int_ [°C]	*T*_g,bulk_ [°C]	*T*_g,int_ [°C]	Δ*T*_g_ [°C]	[V μm^−1^]	[°C]	[μC m^−2^ K^−1^]
E100	27	26	[–]	21	[–]	[–]			
E100-SiO_2_-10%	15	8	23	10	25	15	5	15	0.36 ± 0.26
E100-SiO_2_-50%	[–]	26	17.5	22	4.5				
E100-TiO_2_-10%	13	17	32	22	38	16	2.5	25	1.48 ± 0.48
							2.5	30	2.58 ± 0.80
E100-TiO_2_-50%	[–]	27	20	24.5	4.5				

The addition of SiO_2_ or TiO_2_ nanoparticles in a PDMS matrix has been shown to lower the bulk *T*_g_ (*T*_g,bulk_) of PDMS and, at the same time, lead to an additional glass transition due to the adsorbed polymer chains.^41,43,44^ This interfacial *T*_g_ (*T*_g,int_) is observed at a higher temperature than *T*_g,bulk_ due to the restricted movement of the adsorbed chains. Accordingly, the transition around 8 °C in the E100-SiO_2_-10% can be assigned to *T*_g,bulk_ and around 23 °C to *T*_g,int_, while the neat E100 again shows a single shoulder of the *T*_g,bulk_ at 26 °C. Compared with neat E100, the *T*_g,bulk_ is 18 °C lower in E100-SiO_2_-10%. This shows that adding SiO_2_ particles in the amorphous polar matrix leads to rearranging the polymer chains in the bulk and adsorption at the filler surface. We also observed two shoulders for E100-TiO_2_-10%, albeit at higher temperatures than its SiO_2_ counterpart with a *T*_g,bulk_ of 17 °C, while *T*_g,int_ reaches 32 °C.

The higher-filled composites do not show clear shoulders in TSDC but can be observed in the DRS measurements. Fig. 5d shows the dc-conduction free dielectric loss 
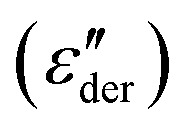
 of the E100-TiO_2_-50% sample plotted as a function of temperature and frequency obtained using the derivative technique introduced by Wübbenhorst and van Turnhout.^73,74^ We can clearly observe the presence of two transitions that shift to higher temperatures with an increase in frequency, pointing to a relaxation process such as glass transition. To confirm this, the derivative curves were subjected to an HN-fit, and the resulting Arrhenius curves are depicted in Fig. 5e. As expected, both these processes show a non-linear relaxation time dependence characteristic of glass transition with an additional relaxation (*α*_int_ relaxation) to the bulk relaxation (*α*_bulk_ relaxation). The corresponding *T*_g_s (at log *τ* = 100 s) are tabulated in Table 1.

Similar to SiO_2_, adding TiO_2_ results in a clear difference in the interfacial *T*_g,int_ values. A strong filler-matrix interaction in E100-TiO_2_-10% results in the *T*_g,int_ to move to 38 °C, which is 13 °C higher than the *T*_g,int_ of E100-SiO_2_-10%. The poorer interaction of SiO_2_ can be seen by the low cooperativity, leading to an almost Arrhenius-like VFT behavior of E100-SiO_2_-10% in Fig. 5e.^50^ In contrast, E100-TiO_2_-10% has a higher cooperativity and a clear VFT behavior (Fig. 5e). The stronger filler–polymer interaction with TiO_2_ compared to SiO_2_ can also be observed in FTIR (Fig. S16, ESI†) and has been observed for PDMS nanocomposites as well.^41,43^ The stronger filler–polymer interaction leads not only to higher *T*_g,int_'s at the TiO_2_ interface but also increases the thickness of the interphase layer.^41^ The polar polymer is adsorbed onto SiO_2_ and TiO_2_ particles of equal size (20 nm diameter) but different surface areas. The SEM images of E100-MO_2_-*x*% are shown in Fig. S17 (ESI†). At low filler loadings, the particles are uniformly distributed in the polymer matrix, such as in the cases of E100-SiO_2_-10% and E100-TiO_2_-10%. As expected, increasing the filler fraction to 50% leads to cluster formation.^41,43^ Cluster formation through the increase in the TiO_2_ in E100-TiO_2_-50% sharply reduces the second *T*_g,int_ to 24.5 °C, close to the *T*_g_ of E100. Similarly, in E100-SiO_2_-50% samples, the cluster formation led to lower interfacial *T*_g,int_ around the *T*_g_ of E100, although the difference between the two *T*_g,int_ of the SiO_2_ composites is smaller compared to the TiO_2_ composites due to the already low layer thickness in the non-clustered SiO_2_ case. The cluster formation reduces the thickness of the interfacial polymer layer and leads to lower cooperativity and lower *T*_g,int_ for both fillers. This aligns with the previously described behavior of cluster formation of SiO_2_ and TiO_2_ in PDMS.^43^ This analysis is further supported by the lower strength of the interfacial process in the 50% filled composites, as seen in Fig. S18 (ESI†).

Concerning the bulk *T*_g_s (*T*_g,bulk_) of the E100-TiO_2_-*x*%, both from Fig. 5e and Table 1, we see similar relaxation time scales and values. Hence, the addition of TiO_2_ affects mainly the *T*_g,int_ but has a minor impact on the *T*_g,bulk_. In accordance with the TSDC results, the addition of SiO_2_ reduces the *T*_g,bulk_. This agrees with previous works showing that highly cooperative filler–matrix interaction has little impact on the *T*_g,bulk_ while low cooperative, weak filler–matrix interaction lowers the *T*_g,bulk_.^75^ An increase in SiO_2_ loading moves its *T*_g,bulk_ in line with the *T*_g,int_ closer to that of E100. Cluster formation consequently reduces the temperature difference between the two *T*_g_s. Interestingly, the difference between the two *T*_g_s is similar for a given weight fraction irrespective of the type of filler with Δ*T*_g_ of 4.5 °C and 15–16 °C for the clustered and non-clustered composites, respectively (Table 1).

To ensure that the two transitions are indeed glass transitions, we further performed a temperature-dependent DMA on the neat E50 as well as the E50 filled with 10 wt% SiO_2_ and TiO_2_, respectively (Fig. S19, ESI†). In line with the dielectric data, the *T*_g,bulk_ shifts downward for the SiO_2_-filled sample and remains constant for the TiO_2_-filled one. The *T*_g,int_ of the TiO_2_ and SiO_2_-filled sample lead to the fact that the rubbery plateau is reached later than the neat E50 sample. The tan(*δ*) shows an unimodal distribution for the unfilled E50 and a bimodal distribution for the composites, leading to the same conclusion as the dielectric data, that two distinct glass transition steps occur. This is further proof of the existence of a interphase layer.

As mentioned before, two *T*_g_s can allow these amorphous nanocomposites to exhibit a remanent polarization between the bulk and interphase *T*_g_s when suitably poled. We investigated the pyroelectric response of the E100-TiO_2_-10% since they had the maximum Δ*T*_g_ as well as the *T*_g,bulk_ below and the *T*_g,int_ well above RT. The samples were polarized above their *T*_g,int_ at 60 °C and cooled down with the poling field (*E*_p_) to a temperature well below *T*_g,int_. After removing the electric field, the pyroelectric coefficient was measured at a temperature (*T*_py_) between the two *T*_g_s, where the oriented dipoles in the adsorbed interphase were frozen. Fig. 5f shows the sample pyroelectric response of E100-TiO_2_-10% at 30 ± 0.5 °C. Since, a sinusoidal temperature change was used, the measured pyroelectric signal is also sinusoidal. For comparison, the pyroelectric response of one day aged E100 and a PDMS-TiO_2_-10% sample at 25 °C is also shown in Fig. 5g. Neither the neat E100 nor the PDMS nanocomposite reference samples exhibit any pyroelectricity. It should be mentioned that the initial measurement of the E100 showed a pyroelectric current, although lower than its TiO_2_-filled counterpart, as shown in Fig. S20 (ESI†). This is because the measurement temperature is near the neat polymer's *T*_g_, which causes the polarization stored in the sample to be gradually lost after poling. In the case of the composite, the polarization in the interfacial layer is frozen at this temperature, and the thermal expansion and contraction of the sample due to the applied thermal stress, the dipole density of the composite changes, leading to a secondary pyroelectric current.

Consequently, the rise of pyroelectricity in these systems results from the two *T*_g_s arising from the filled system in combination with these *T*_g_'s occurring around RT. This is the first time this strategy has been used to turn fully amorphous homopolymers pyroelectric. The pyroelectric current does not occur in the neat and aged E100 nor an analogous composite made from 10 wt% TiO_2_ in PDMS. Even at a very low *E*_p_ = 2.5 V μm^−1^, E100-TiO_2_-10% shows a higher *p*-coefficient at 30 °C compared to other amorphous-based pyroelectric polymers poled at much higher fields reported in the literature.^76^ This can be attributed to the high permittivity of the E100 polar polymer, a high thermal coefficient of expansion combined with the polarity of TiO_2_ particles, which results in a strongly adsorbed interfacial layer.^77–79^ From a practical point of view, the E100-TiO_2_-10% shows the highest interfacial *T*_g_ of 38 °C, indicating its suitability for use in RT applications. The aging behavior of such a composite at 25 and 30 °C was studied to assess the stability of its pyroelectric response (Fig. 5h). After 41 days, a *p*-value of 3.4 μC m^−2^ K^−1^ was measured at 30 °C, which was 87% of the value measured on the freshly measured sample. On the other hand, a *p* = 0.97 μC m^−2^ K^−1^ was measured in the same sample at 25 °C, which was 45.5% of the original value. The lower *p*-coefficient at 25 °C might be explained by the proximity to the *T*_g,bulk_ at 22 °C (Fig. 5i). At 25 °C bulk phase is still partially glassy, reducing the overall expansion. In contrast, at 30 °C, the bulk process is largely completed, leading to stronger thermal expansion. The expansion would be even higher at 35 °C, but here, the stability of the interphase will be reduced as the temperature approaches *T*_g,int_. These phenomena can also explain the lower stability of the original *p*-coefficient at 25 °C, as in the initial measurement, a higher number of dipoles in the bulk were still aligned, and the orientation was gradually lost until the bulk had no orientation anymore. In contrast, at 30 °C, the bulk orientation is already in the first measurement, largely lost, leading to only a 13% decrease in *p*-coefficient. Hence, we can conclude that E100-TiO_2_-10% shows pyroelectric with remarkable stability at 30 °C, allowing it to be used as a pyroelectric sensor or for micro-energy harvesting applications. It appears reasonable to expect that fillers with even higher polarity could lead to even higher *T*_g,int_ values and thicker layers, which would increase the operation window of the composites as well as the *p*-coefficient.

## 3. Conclusions

We synthesized four novel polysiloxanes modified with cyanopropyl and nitroaniline polar side groups, exhibiting an 8- to 12-fold increase in permittivity compared to regular PDMS. The material containing the highest concentration of nitroaniline has a glass transition temperature of 27 °C and a relative permittivity of 35 at 40 °C. To our knowledge, this permittivity is the highest reported permittivity of an amorphous neat elastomer. We reached the highest values possible by grafting dipoles onto the polymer backbone. Increases in permittivity are accompanied by significant shifts in *T*_g_s, creating a trade-off between elasticity and permittivity, as thermal expansion above the *T*_g_ reduces relative permittivity but improves elasticity. We propose *T*_g_ + 60 °C as a universal guideline for designing elastomers that balance high relative permittivity with good elasticity (tan(*δ*) ≤ 0.1).

The high permittivity and the near room temperature *T*_g_ of the nitroaniline-modified polysiloxane are exploited for use in pyroelectric applications by introducing SiO_2_ and TiO_2_ nanofillers. Adding fillers results in an interfacial adsorbed polymer layer *via* hydrogen bonding, which shows a delayed dielectric response compared to the bulk polymer, resulting in an additional *T*_g_ above its bulk *T*_g_. Hence, poling the nanocomposites above their interfacial *T*_g_ and cooling it with the field to a temperature between the two *T*_g_s, will result in a quasi-polarized system that exhibits pyroelectricity. Two weight fractions of SiO_2_ and TiO_2_ fillers (10 and 50 wt%) were added, and their dielectric behavior was studied. Due to its polarity, samples with 10 wt% of TiO_2_ show a strong filler–matrix interaction that pushes its interfacial *T*_g_ to 38 °C. In addition, it can lead to an increased local field in the interfacial layer. This, in turn, leads to a strong and stable pyroelectric response near room temperature at low-poling fields. After aging the sample for 41 days, a pyroelectric coefficient of 3.4 μC m^−2^ K^−1^ is recorded for a 10 wt% TiO_2_ composite at 30 °C, which is higher than other amorphous-based pyroelectric materials reported in the literature.

## 4. Experimental section

### 4.1. Materials

1,3,5,7-Tetramethylcyclotetrasiloxane (D_4_H_4_), TMAH (40% wt in water), Karstedt's catalyst (platinum(0)-1,3-divinyl-1,1,3,3-tetramethyldisiloxane complex solution in xylene, Pt ≈ 2%) (Pt-cat), allyl cyanide, divinyldimethylsilane, hydrophobic (amorphous, hexamethyldisilazane treated, 20 nm) SiO_2_ were purchased from ABCR. *N*-Methyl-4-nitroaniline, allyl alcohol, palladium-(II)-acetate, triphenylphosphine, titanium-(IV)-isopropoxide, *o*-xylene, dry toluene, and titanium dioxide (Aeroxide P25, 20 nm) were purchased from Sigma-Aldrich. Ethyl acetate, heptane, toluene, and tetrahydrofuran were purchased from VWR. *N*-Allyl-*N*-methyl-4-nitroaniline was prepared according to the literature.^37^ All chemicals were of reagent grade and used without purification.

### 4.2. Methods

Information about material characterizations can be found in the ESI.†

#### 4.2.1. Synthesis of monomers M100, M75, M50, and M25

All monomers were prepared by a one-pot hydrosilylation reaction under an argon atmosphere (Fig. 1a). To a solution of D_4_H_4_ (2 g, 2.03 mL, 8.32 mmol) in dry toluene (20 mL), different amounts of *N*-allyl-*N*-methyl nitroaniline were added (8 g for monomer M100, 4.8 g for monomer M75, 3.2 g for monomer M50, 1.6 g for monomer M25). After stirring the solution for 5 min at 80 °C, Karstedt's catalyst (0.2 mL) was added. The reaction mixture was stirred at 110 °C under reflux until the full conversion of *N*-allyl-*N*-methyl nitroaniline. In the case of monomer M100, until the D_4_H_4_ was completely functionalized. For the synthesis of monomers M75, M50, and M25, allyl cyanide was added in excess to the reaction mixture (1.5 g for monomer M75, 2.5 g for monomer M50, 3.6 g for monomer M25) and refluxed at 110 °C for 2 days until complete functionalization. Afterwards, the solvent was distilled, and the product was purified by column chromatography with varying concentrations of heptane/ethyl acetate (1 : 1) as eluent. Monomers M100, M75, M50, and M25, with conversions between 81–94% were synthesized. It should be noted that with the exception of M100, the other monomers have an average mol% of 75, 50, and 25% NA in their composition.

Monomer M100: ^1^H NMR (400 MHz, CDCl_3_, *δ*): 0.10 (m, 12H, SiCH_3_–O–), 0.50 (t, 8H, Si–CH_2_–), 1.61 (m, 8H, –CH_2_–CH_2_–), 3.02 (m, 12H, –N–CH_3_), 3.35 (m, 8H, –CH_2_–N–), 6.54 (d, 8H, H_Ar_(f)), 8.06 (d, 8H, H_Ar_(g)) (Fig. 1c); ^13^C NMR (100 MHz, CDCl_3_, *δ*): −0.51 (SiCH_3_–O–), 14.07 (Si–CH_2_–), 20.14 (–CH_2_–CH_2_–), 38.94 (N–CH_3_), 55.35 (–CH_2_–N–), 110.28 (C_Ar_(f)), 126.20 (C_Ar_(g)), 136.92 (C_Ar_–NO_2_), 153.17 (C_Ar_–N) (Fig. S21a, ESI†).

Monomer M75: ^1^H NMR (400 MHz, CDCl_3_, *δ*): 0.10 (m, 12H, SiCH_3_–O–), 0.50 (t, 8H, Si–CH_2_–), 1.65 (m, 8H, –CH_2_–CH_2_–), 2.36 (m, 4H, –CH_2_–CN), 3.05 (m, 6H, –N–CH_3_), 3.40 (m, 4H, –CH_2_–N–), 6.62 (d, 4H, H_Ar_(f)), 8.11 (d, 4H, H_Ar_(g)) (Fig. S2, ESI†); ^13^C NMR (100 MHz, CDCl_3_, *δ*): −0.75 (SiCH_3_–O–), 14.01 (Si–CH_2_–), 20.10 (–CH_2_–CH_2_–CN), 20.30 (–CH_2_–CH_2_–NA), 38.77 (N–CH_3_), 55.26 (–CH_2_–N–), 110.20 (C_Ar_(f)), 118.16 (–C≡N); 126.20 (C_Ar_(g)), 136.87 (C_Ar_–NO_2_), 153.10 (C_Ar_–N) (Fig. S21b, ESI†).

Monomer M50: ^1^H NMR (400 MHz, CDCl_3_, *δ*): 0.10 (m, 12H, SiCH_3_–O–), 0.50 (t, 8H, Si–CH_2_–), 1.65 (m, 8H, –CH_2_–CH_2_–), 2.36 (m, 4H, –CH_2_–CN), 3.05 (m, 6H, –N–CH_3_), 3.40 (m, 4H, –CH_2_–N–), 6.62 (d, 4H, H_Ar_(f)), 8.11 (d, 4H, H_Ar_(g)) (Fig. 1c); ^13^C NMR (100 MHz, CDCl_3_, *δ*): −0.56 (SiCH_3_–O–), 16.47 (Si–CH_2_–), 19.78 (–CH_2_–CH_2_–CN), 20.44 (–CH_2_–CH_2_–NA), 39.65 (N–CH_3_), 55.68 (–CH_2_–N–), 110.96 (C_Ar_(f)), 119.71 (–C≡N); 126.37 (C_Ar_(g)), 126.51 (C_Ar_–NO_2_), 152.99 (C_Ar_–N) (Fig. S21c, ESI†).

Monomer M25: ^1^H NMR (400 MHz, CDCl_3_, *δ*): 0.10 (m, 12H, SiCH_3_–O–), 0.50 (t, 8H, Si–CH_2_–), 1.65 (m, 8H, –CH_2_–CH_2_–), 2.36 (m, 4H, –CH_2_–CN), 3.05 (m, 6H, –N–CH_3_), 3.40 (m, 4H, –CH_2_–N–), 6.62 (d, 4H, H_Ar_(f)), 8.11 (d, 4H, H_Ar_(g)) (Fig. S2, ESI†); ^13^C NMR (100 MHz, CDCl_3_, *δ*): −0.65 (SiCH_3_–O–), 16.33 (Si–CH_2_–), 19.67 (–CH_2_–CH_2_–CN), 20.40 (–CH_2_–CH_2_–NA), 39.20 (N–CH_3_), 55.63 (–CH_2_–N–), 110.96 (C_Ar_(f)), 119.46 (–C≡N); 126.31 (C_Ar_(g)), 132.48 (C_Ar_–NO_2_), 168.27 (C_Ar_–N) (Fig. S21d, ESI†).

#### 4.2.2. Synthesis of the cross-linker

The synthesis was performed according to our previously reported synthesis.^33^ In a 500 mL three-neck flask equipped with a magnetic stirrer and a dropping funnel, D_4_H_4_ (54 mL, 222.5 mmol) was dissolved in *o*-xylene (100 mL) and mixed for 5 min. Karstedt's catalyst (0.1 mL) and divinyldimethylsilane (13.5 mL, 89 mmol) were dissolved in *o*-xylene (100 mL) and the mixture was slowly added to the D_4_H_4_ solution. After addition, the reaction was stirred at RT for 24 h. Then, the excess D_4_H_4_ was removed *in vacuo*. Allylcyanide (50.4 mL, 623 mmol) and Karstedt's catalyst (0.1 mL) were added to the reaction mixture, and the mixture was stirred for 4 days at 110 °C. The solvent was removed in a rotary evaporator, and the product was dried in a high vacuum.

^1^H NMR (400 MHz, CDCl_3_, *δ*): −0.03 (s, 6H), 0.14 (m, 35H), 0.42 (s, 8H), 0.73 (m, 12H), 1.71 (m, 12H), 2.39 (m, 12H) (Fig. S22, ESI†). ^13^C NMR (100 MHz, CDCl_3_, *δ*): −4.10 (Si–CH_3_), −0.21 (SiCH_2_), −0.29 (SiCH_3_–O–), 16.85 (Si–CH_2_), 20.09 (–CH_2_–CH_2_–), 20.81 (–CH_2_–CN), 120.19 (–C≡N) (Fig. S23, ESI†).

#### 4.2.3. Synthesis and processing of polymers P100, P75, P50, P25 and elastomers E100 and E50

The polymers were synthesized as previously reported.^33,61^ In short, 2 g of monomer was polymerized with 2 μL of TMAH 40 wt% in water. The TMAH was previously dried under a vacuum to remove the water. The monomers were then polymerized at 110 °C for 30 min followed by 2 h at 60 °C, leading to polymers P100, P75, P50, and P25.

Monomer (M100 or M50) (1.8 g) and cross-linker (0.2 g) were polymerized with TMAH 40 wt% in water (2 μL). The TMAH was previously dried under a vacuum to remove the water. The mixture was then polymerized at 110 °C for 30 min followed by 2 h at 60 °C, leading to polymers elastomers E100 and E50. Films of E100 and E50 were made inside a metal frame with a thickness of 250 μm and enclosed between two Teflon substrates. Thinner layers were melt-pressed without a frame.

#### 4.2.4. Synthesis and processing of composites E100 with TiO_2_ and SiO_2_

Monomer M100 (1.8 g) and cross-linker (0.2 g) were polymerized with TMAH 40 wt% in water (2 μL). The TMAH was previously dried under a vacuum to remove the water. To achieve the 50 wt% filled composites to a mixture of monomer and cross-linker (2 g), the filler (2 g) was added. For the 10 wt% filler, 0.23 g filler was added to 2 g of polymerization mixture. All components were thoroughly mixed by a planetary mixer (Speedmixer) at 3500 rpm for 10 minutes. After the mixing, the samples were five times three-roll-milled to ensure good particle dispersion. The samples were placed in a melt-pressed at 110 °C for 30 minutes, followed by 60 °C for 2 h at a pressure of 1 ton without a frame. During this time, the polymerization and cross-linking occurred.

## Conflicts of interest

There are no conflicts to declare.

## Supplementary Material

MH-012-D5MH00234F-s001

## Data Availability

The data that support the findings of this study are available in the ESI,† of this article and the raw data used to generate the figures can be found at https://doi.org/10.5281/zenodo.14764813.
