# Supplementary material for: Pushing the boundaries of dielectric permittivity in polysiloxanes: polar dipole modifications enable amorphous pyroelectric polymers
Source: Mater Horiz. 2025 Apr 21;12(14):5287–301. doi: 10.1039/d5mh00234f (PMC12047071; doi:10.1039/d5mh00234f)
Supplement: MH-012-D5MH00234F-s001 [file MH-012-D5MH00234F-s001.pdf]

ARTICLE

Supporting Information

Pushing the boundaries of dielectric permittivity in polysiloxanes: polar dipole modifications enable amorphous pyroelectric polymers

*Patrick M. Danner, Thulasinath Raman Venkatesan, Johannes von Szczepanski, Francis Owusu, Dorina M. Opris**

Tensile measurements: Tensile measurements were performed with a Zwick Z010 tensile test

machine with a 50 mm min^{-1} speed using dog-bone-shaped samples of 18 mm length and 2 mm width. The Young's moduli were determined from the linear fit slope of the stress–strain curves from 0 ≤ 10%.

Dynamic mechanical analysis: DMA was carried out on an RSA 3 DMA from TA Instruments in the tensile mode. Stripes of samples with rectangular geometry (10 mm × 30 mm) were measured under a dynamic load of 2 g. Data in the frequency sweeps were obtained by applying 2% strain in the frequency range of 0.05–10 Hz. The amplitude scans were performed at 0.1 Hz from 0.05% to 10% strain. The temperature sweeps were performed in the temperature range indicated in each measurement.

Broadband Dielectric Spectroscopy: Temperature-dependent dielectric properties were evaluated using a Novocontrol Alpha-A Frequency Analyzer equipped with quatro cryosystem temperature control. Dielectric spectra were recorded by applying an external voltage of 1 V in an AC frequency range of 0.01 Hz to 1 MHz at RT and in a specified temperature range.

Dielectric Breakdown Measurement: A 12.5 kV DC high voltage power supply system (HCL 35–12500 pos, *FUG electronic GmbH*, Germany) was used for all measurements. Samples were sandwiched between two metal electrodes with 1 mm^2 area embedded in an epoxy resin. The applied voltage was linearly increased until failure voltage was recorded on several samples. Statistical analysis was employed to evaluate the dielectric breakdown field, commonly following the Weibull probability model.
Actuation tests: Electromechanical tests were performed with circular membrane actuators. The polymer films were fixed between two circular frames after applying a biaxial pre-stretch of 7.4%. Carbon black powder was applied on each film side with a brush as circular electrodes. The electrodes were connected to a FUG HCL-35-12500 HV power supply with aluminum foil. The actuation strain was determined optically with a digital camera detecting the edge between the black electrode and the light silicone film, thus measuring the extension of the electrode diameter. The extension was measured at two different positions and the values were averaged.
NMR spectroscopy: ^1H and ^{13}C NMR spectra were recorded at 298 K on a Bruker Avance 400 NMR spectrometer using a 5 mm broadband inverse probe at 400.13 and 100.61 MHz, respectively. Chemical shifts (δ) in ppm are calibrated to residual solvent peaks ($CDCl_3$: δ = 7.26 and 77.16 ppm)

Please do not adjust margins

Gel permeation chromatography (GPC): Gel permeation chromatograms were recorded using an Agilent 1100 Series HPLC (columns: serial coupled PSS SDV 5 m, 100 Å and PSS SDV 5 m, 1000 Å, detector: DAD, 235 nm and 360 nm; refractive index), where THF was the mobile phase. Poly(methyl methacrylate) (PMMA) was used as standard.

Differential scanning calorimetry (DSC): DSC investigations were undertaken on a PerkinElmer Pyris Diamond instrument under a nitrogen flow (50 mL min^{-1}) in aluminum crucibles, which were shut with pierced lids and contained about 10 mg sample mass. Two heating and one cooling steps with a heating and cooling rate of 20 K min^{-1} were conducted per measurement under a nitrogen flow (50 mL min^{-1}). The second heating step was considered for the evaluation of the T_g.

Thermalgravimetric analysis (TGA): TGA was performed with a *Netzsch* TG 209-F1 with vacuum-tight thermo-microbalance. Samples were measured in an Al_2O_3 crucible at a heating rate of 20 K min^{-1} under nitrogen flow.

Scanning electron Microscopy (SEM): SEM images were recorded on a FEI Quanta 650 ESEM. Images were taken in secondary electrons-topography mode using Everhart–Thornley Detector at 20 kV acceleration voltage.

Thermally Stimulated Depolarization Currents (TSDCs): A Keysight B2985A electrometer with a built-in DC voltage source was used to both pole and measure the depolarization currents. The composite film was poled at a (T_p) of 50 °C for 10 min before cooling it to -20 °C under the applied field before the start of TSDC measurements. The films were heated at 5 K min^{-1} using a Novocontrol Quatro cryosystem under a dry nitrogen atmosphere.

Fourier-Transform InfraRed (FTIR) spectroscopy: FTIR measurements were made at RT in Attenuated-Total-Reflection (ATR) mode using a Bruker 27 FTIR spectrometer.

Pyroelectric measurements: The quasi-static pyroelectric coefficient (p) was measured by applying a sinusoidal temperature variation to a poled nanocomposite film using a Novocontrol Quatro cryosystem. A modulation frequency of 8.3 mHz, a mean temperature amplitude of 1 K and a mean temperature of 1 °C were used for the measurements. The resulting current was measured using a Keysight B2985A electrometer.

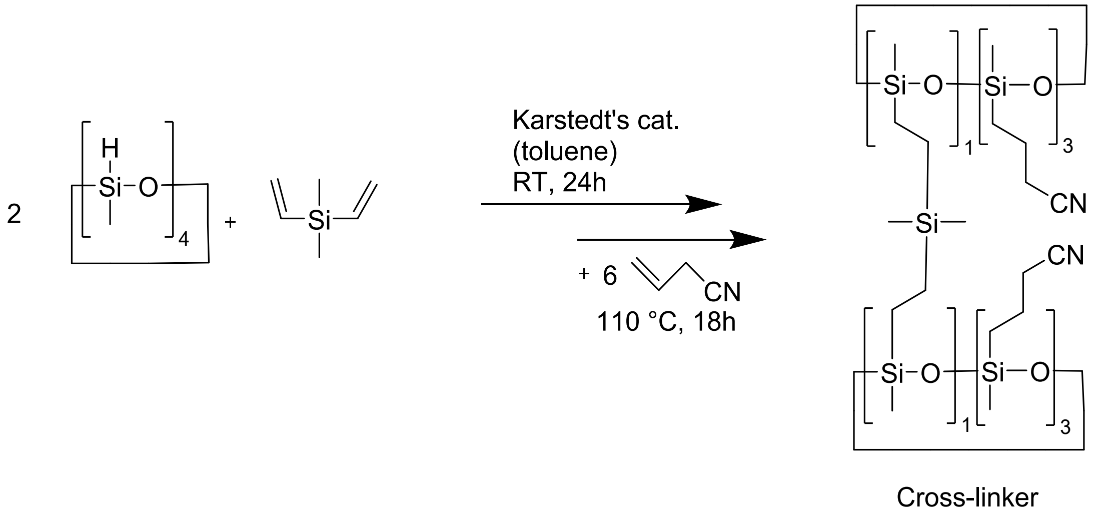

Figure S1. a) One-pot synthesis of polar cross-linker modified with cyanopropyl groups. Polymerization of the four monomers with the cross-linker leads to elastomer networks as the anionic ring-opening polymerization (AROP) initiated by TMAH opens the monomers and cross-linker rings. The cross-linking leads to a dynamic network that can polymerize and depolymerize triggered by temperature.

This journal is © The Royal Society of Chemistry 20xx

Please do not adjust margins

Please do not adjust margins

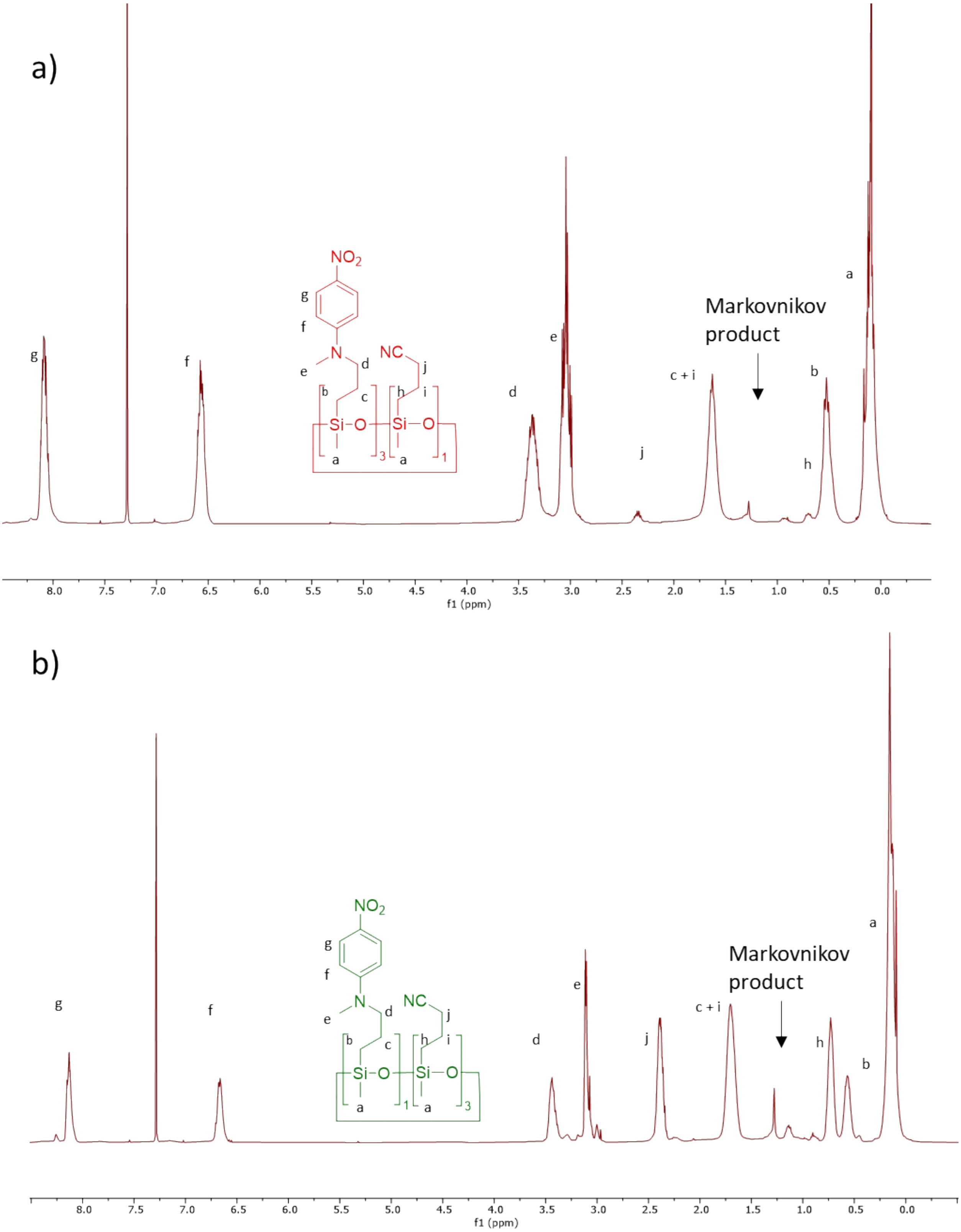

Figure S2. ^{1}H NMR of a) monomer **M75** and b) monomer **M25** in $CDCl_3$.

Please do not adjust margins

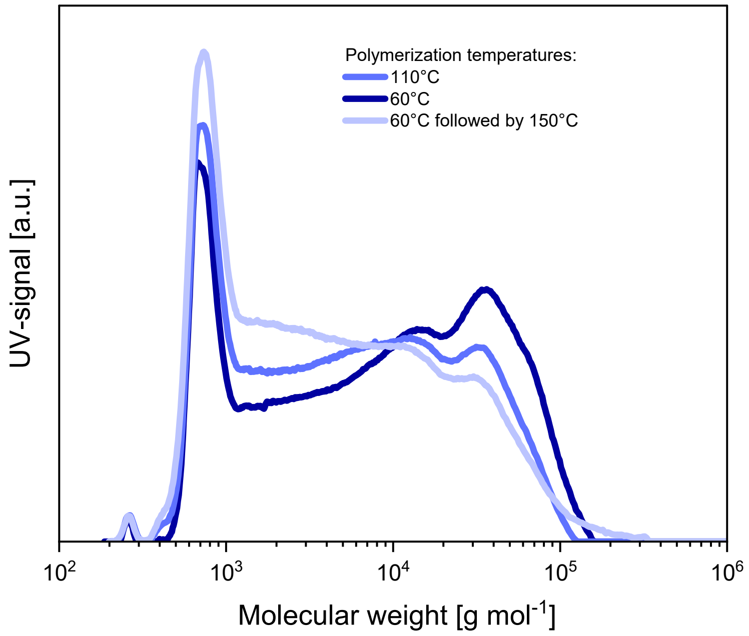

Figure S3. Testing of polymerization temperatures and catalyst deactivation for the polymerization of the monomer **M50** to polymer **P50**. The first test was performed polymerization for 2 h at 110 °C. The second test was performed for 30 minutes at 110 °C followed by 60 °C for 2 h. The last test was performed for 30 minutes at 110 °C followed by 60 °C for 2 h and, lastly, 30 minutes at 150 °C to deactivate TMAH. The deactivation of the catalyst leads to shifting the equilibrium toward the monomer and significant depolymerization. The catalyst in the other two polymers was deactivated by washing three times with water.

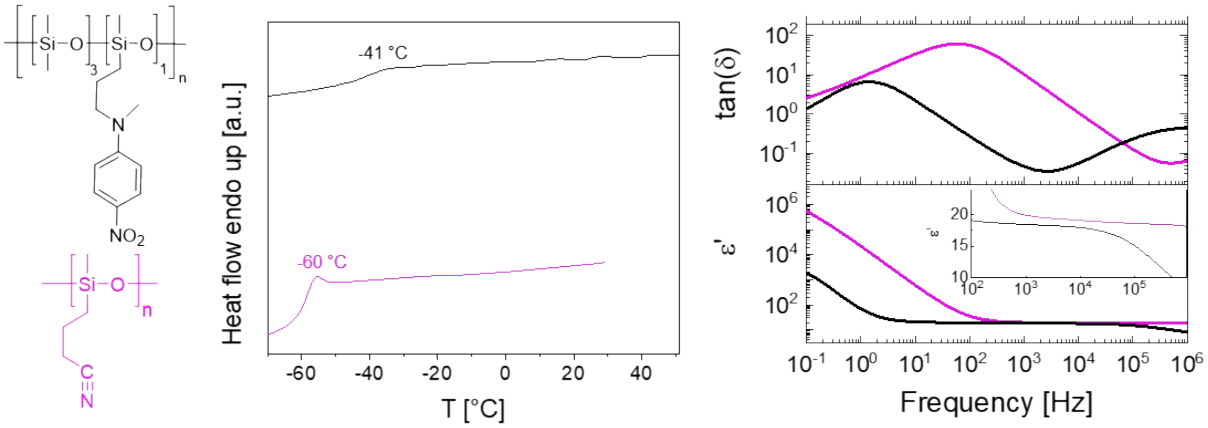

Figure S4. Previously reported polymers from our group serve as a comparison for the four reported polymers **P100**, **P75**, **P50**, and **P25**.[34,61]

This journal is © The Royal Society of Chemistry 20xx

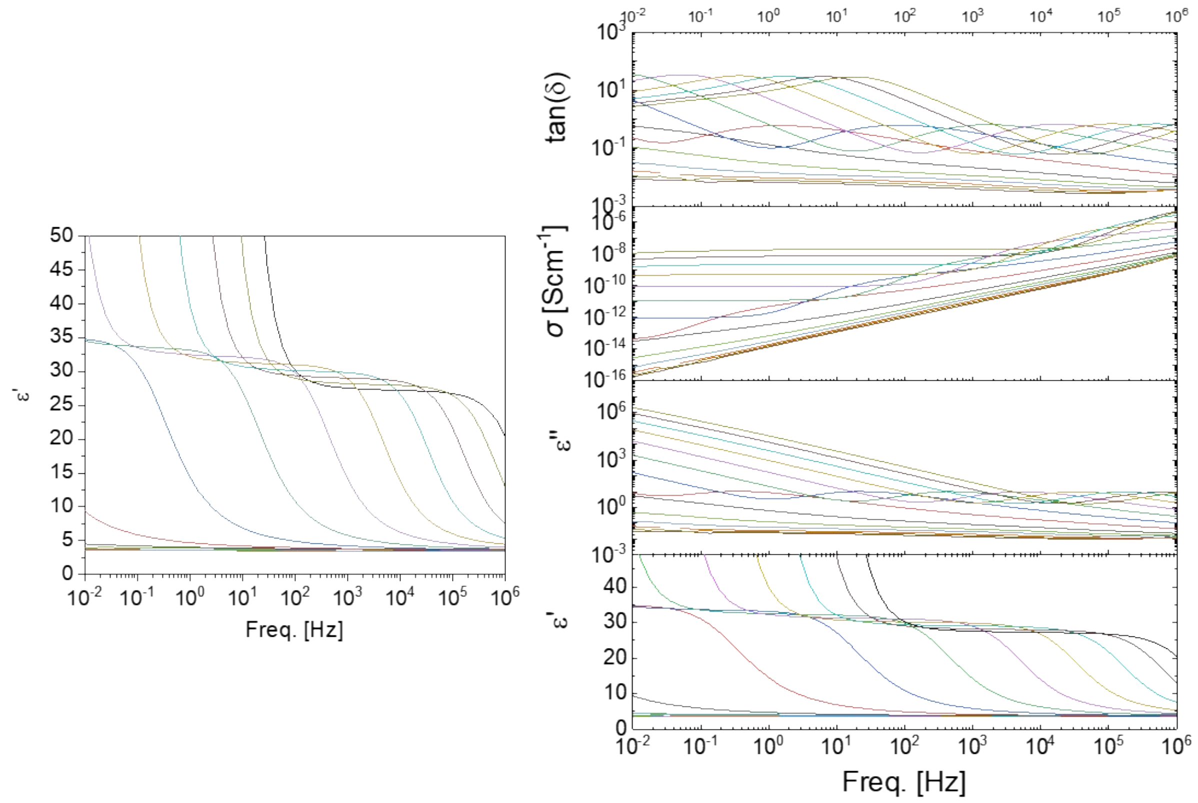

Figure S5. Dielectric spectroscopy measurements of **P100** were conducted from -30 °C to +100 °C in 10 °C increments.

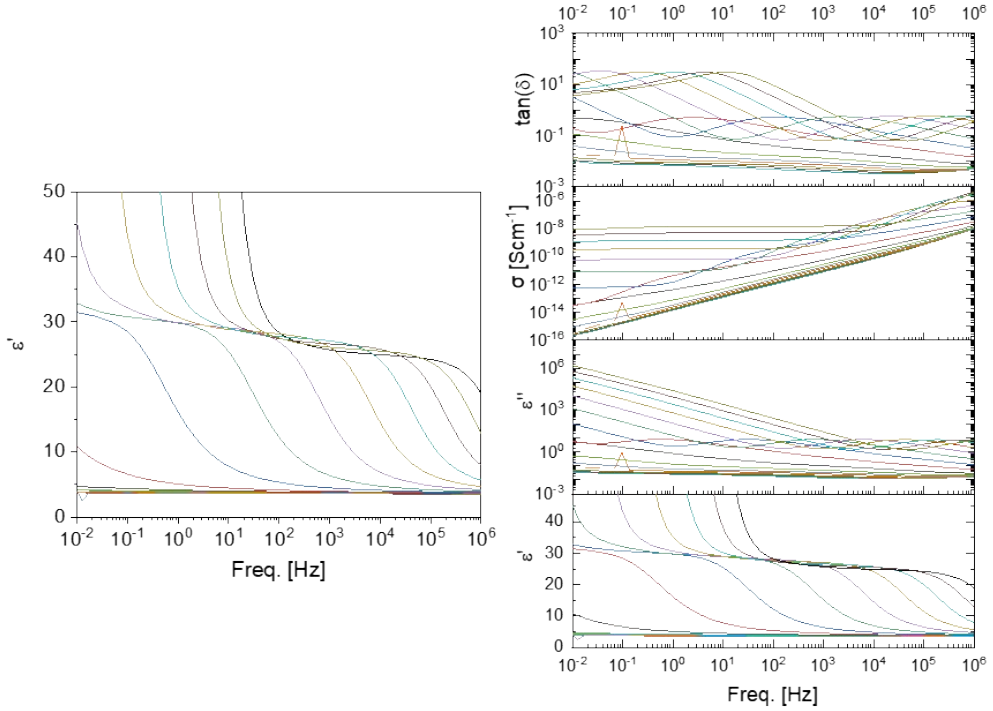

Figure S6. Dielectric spectroscopy measurements of **P75** -30 °C to +80°C in 10°C intervals.

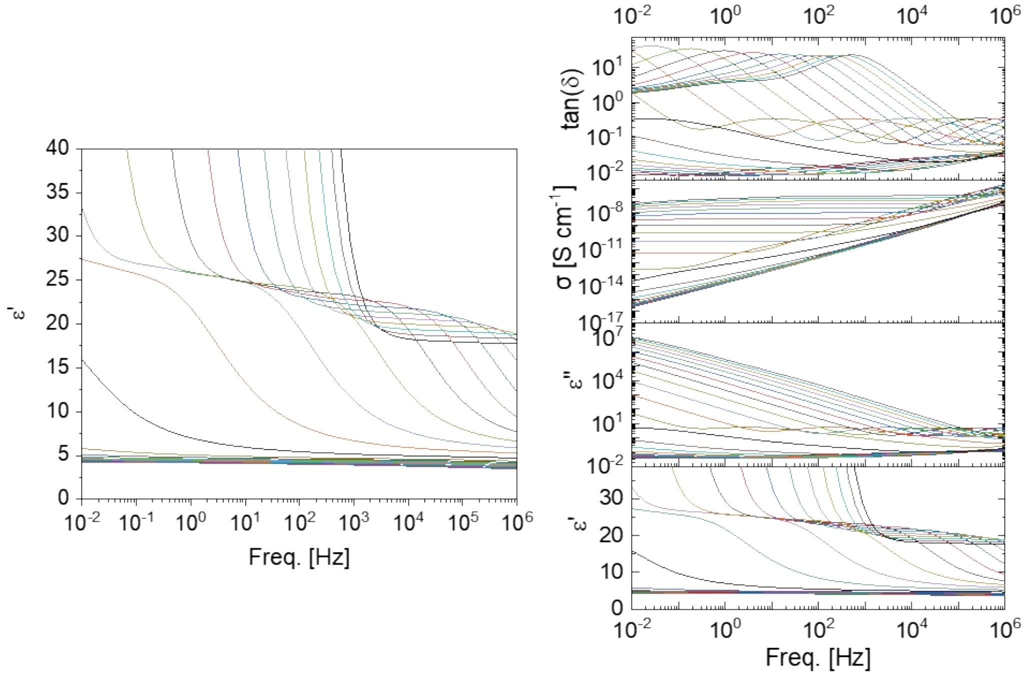

Figure S7. Dielectric spectroscopy measurements of **P25** were conducted from -100 °C to +100 °C in 10 °C increments.

 This journal is © The Royal Society of Chemistry 20xx

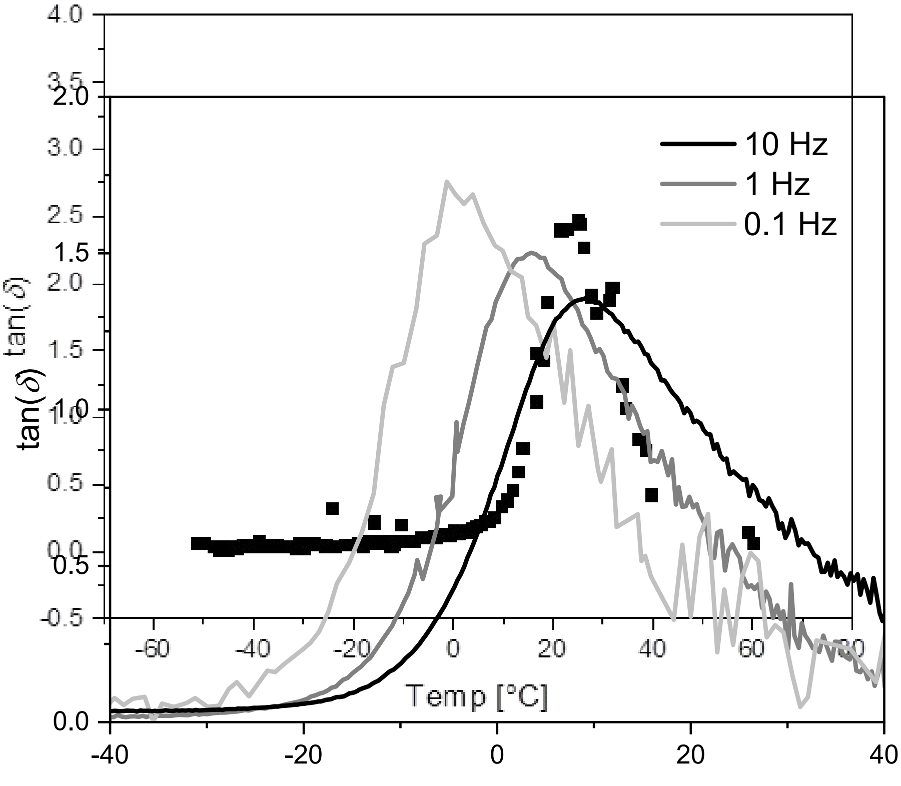

Figure S 8. Temperature sweep DMA measurement showing the loss tangent of the **E100** at 0.1 Hz. The peak is at 25.4 °C.

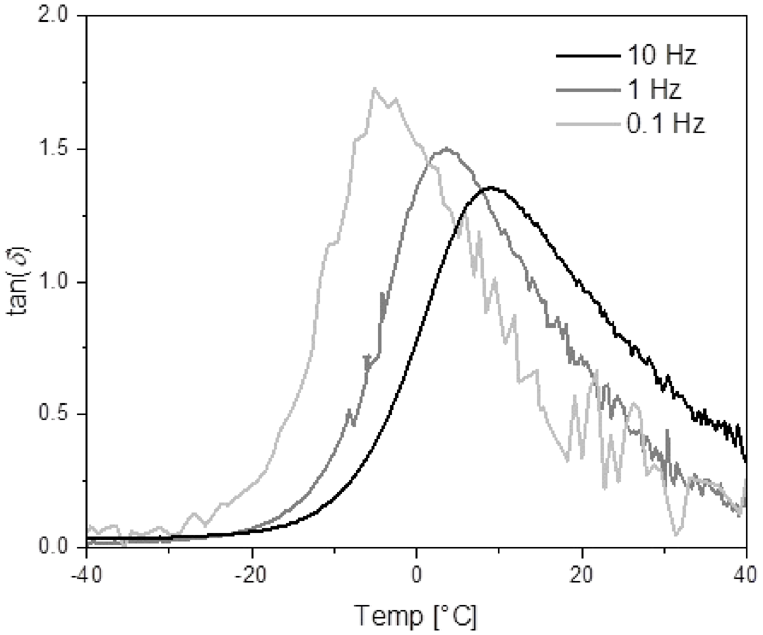

Figure S 9. Temperature sweep DMA measurement showing the loss tangent of the **E50** at 0.1. 1 and 10 Hz. The peak is at -2.5 °C, 3.5 °C and 9.1 °C, respectively.

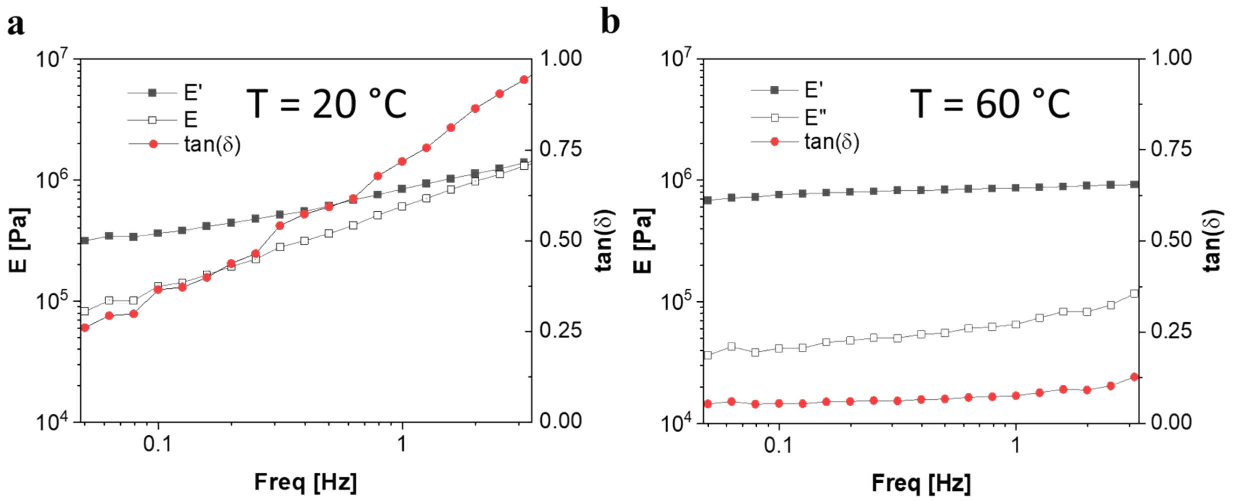

Figure S10. DMA of elastomer **E50**: a) Frequency sweep of the elastomer **E50** at 1% strain at 20 °C b) Frequency sweep of elastomer **E50** at 1% strain at 60 °C.

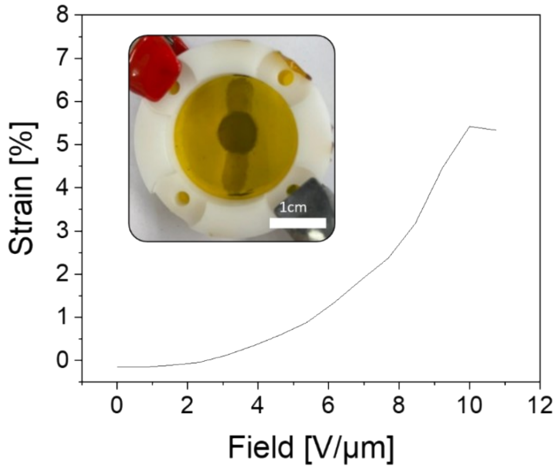

Figure S11. DEA made from elastomer **E50**.

This journal is © The Royal Society of Chemistry 20xx

Please do not adjust margins

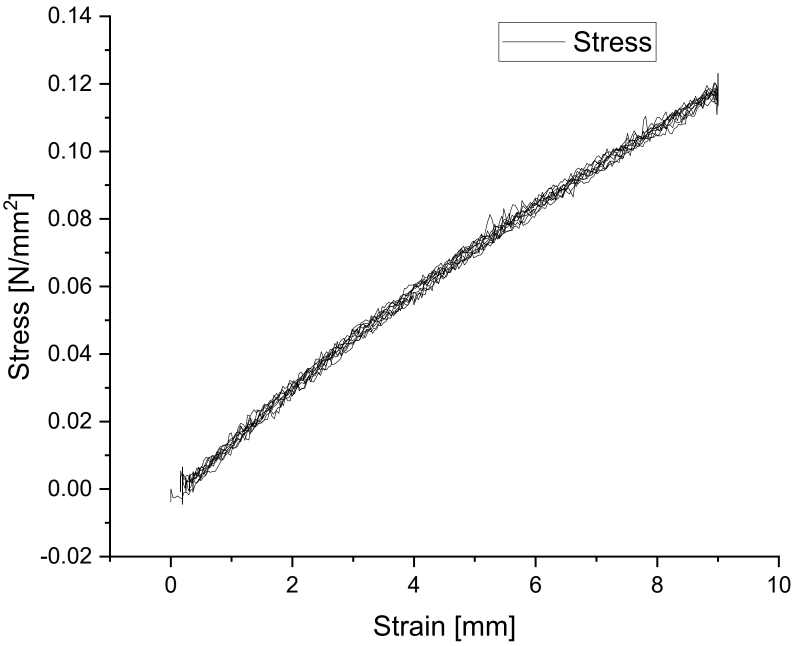

Figure S 12. Cyclic stress-strain curve of E50 with 5 cycles at a speed of 50 mm min^{-1}.

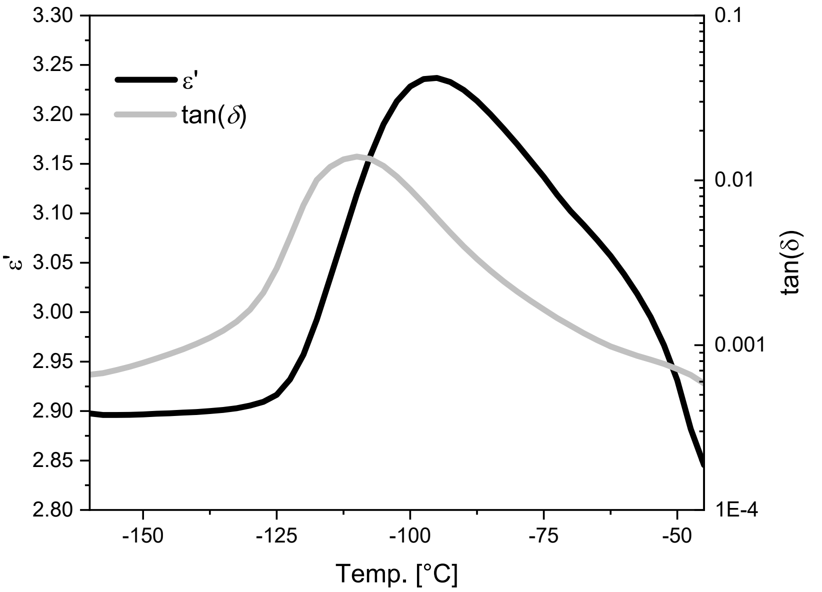

Figure S13. Relative permittivity and dielectric loss factor of PDMS between -160 and -45°C. The measurement was performed on a 100 µm thick sample with an AC voltage of 1V and a frequency of 1k Hz. The peak of the tan(δ) gives the glass transition of the polymer at -110 °C. The maximum relative permittivity is reached at -95 °C and starts decreasing afterward. The T_g+60 °C guideline would place the ideal operating temperature at this frequency at -50 °C. This does indeed coincide with the end of the glass-transition process indicated by the flattening of the tan(δ) curve.

This journal is © The Royal Society of Chemistry 20xx

Please do not adjust margins

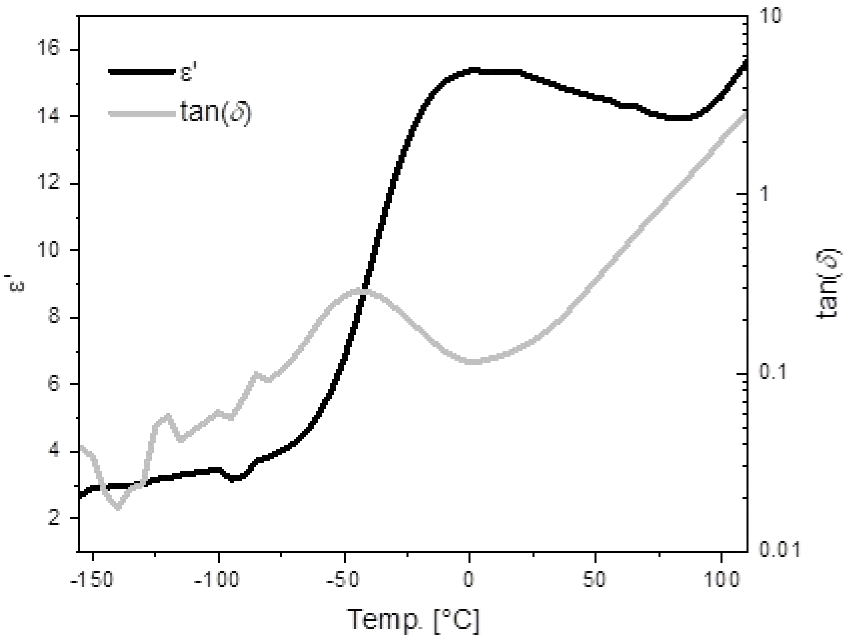

Figure S14. Relative permittivity and dielectric loss factor of 3-cyanopropyl-methyl-polysiloxane from -155 °C up to +110°C. The measurement was performed on a 100 µm thick sample with an AC voltage of 1V and a frequency of 1M Hz. The high frequency was selected to eliminate electrode polarization. The peak of the tan(δ) gives the glass transition of the polymer at -40 °C. The maximum relative permittivity is reached at 0 °C and starts decreasing afterward. The onset of electrode polarization can be observed by the increase of permittivity above 80°C. The T_g+60 °C guideline would place the ideal operating temperature at this frequency at 20 °C. At 20 °C, the glass-transition process is over-indicated by the local minimum of the tan(δ) curve.

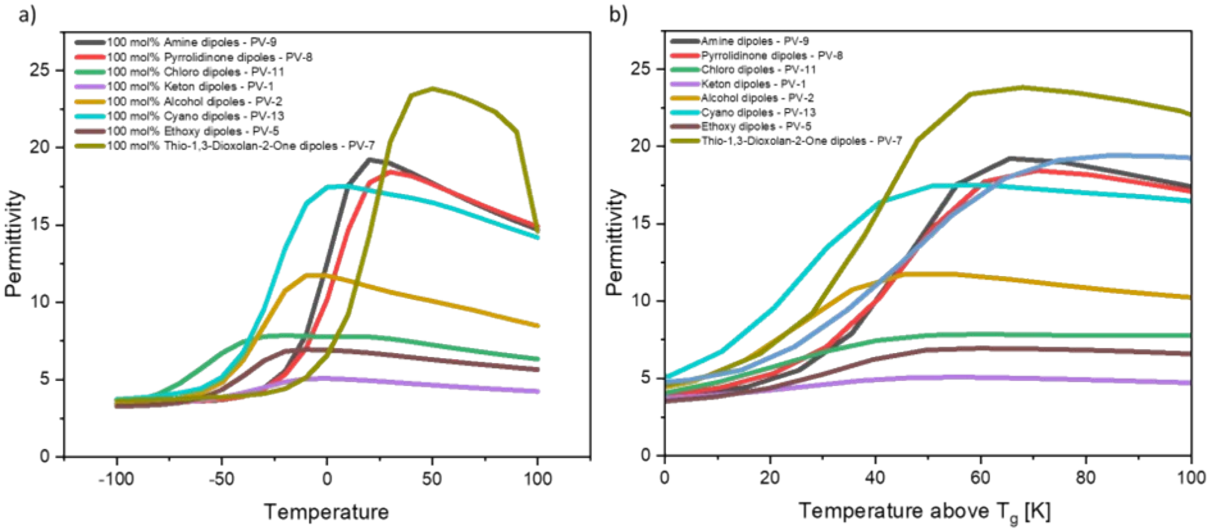

Figure S15. Reference polar polymers from Sheima et al.[22] a) Permittivity vs T_g b) T_g values are set to 0 to identify the peak and decrease behavior. All polymers show a peak in permittivity around 60 °C ± 10 °C followed by a drop in permittivity. PV-X is the number given by the original publication and is given for easier reference.

Please do not adjust margins

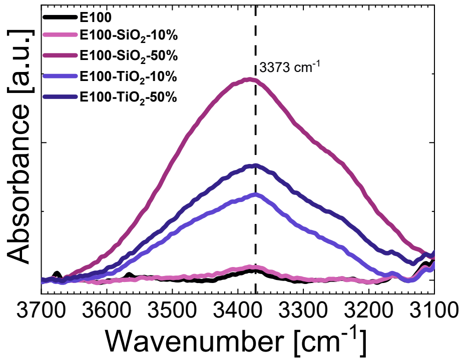

Figure S 16. FTIR spectra between 3700 cm^{-1} and 3100 cm^{-1} of the neat polar matrix and the metal oxide composites. The absorption leads to a broad peak around 3373 cm^{-1}, which can be assigned to intermolecular H-bonding due to the adsorption of the interfacial polymer layer. The adsorption of the polymer chain onto the metal oxides is expected to primarily occur via the hydrogen bonding interactions between the –OH groups found on the surface of the filler and the oxygen atoms in the polar polymer.[43,52,77,78] The broad nature of the peak is characteristic of such bounded groups. These results agree with the previous findings that the polymer-filler interactions are stronger in the case of TiO_2 than SiO_2[41,43,79] as we observe a strong absorption in FTIR even for a lower TiO_2 fraction (10 wt.%) and is attributed to the semi-conducting nature of TiO_2.[43]

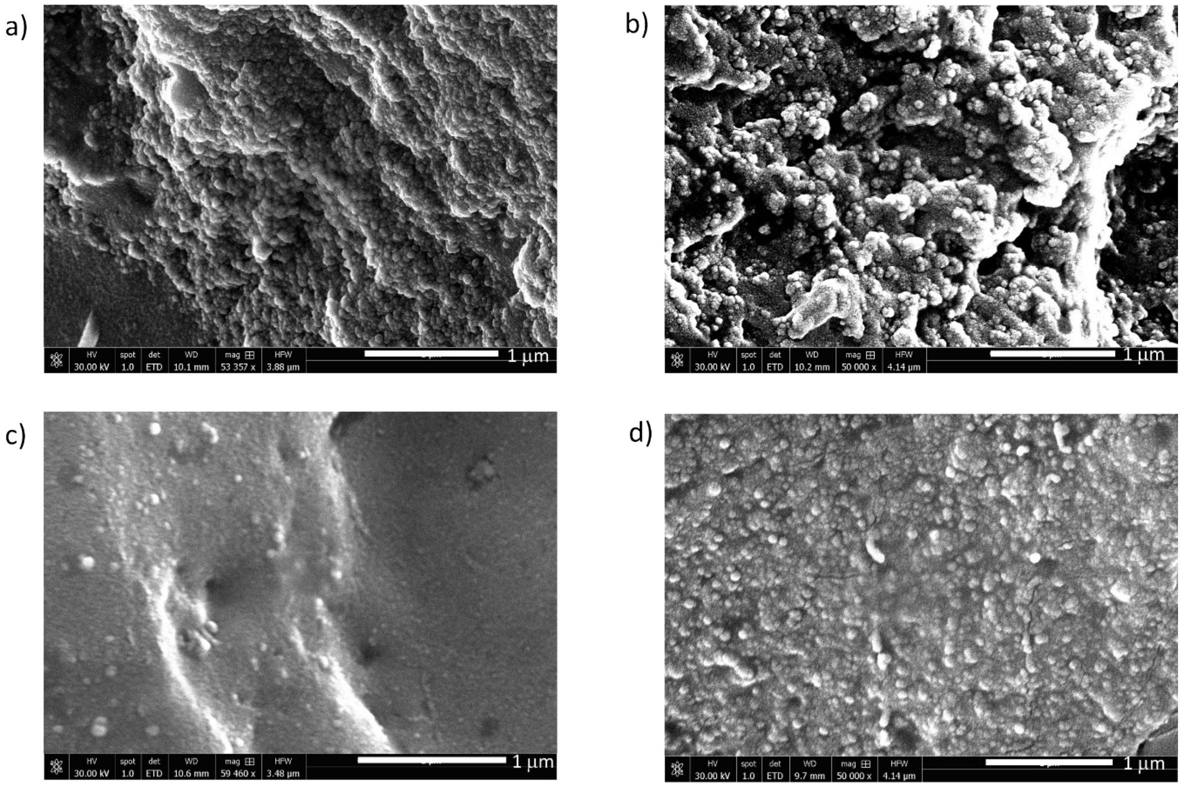

Figure S 17. SEM images of the composites form **E100**. a) **E100-SiO_2-10%**. b) **E100-SiO_2-50%**. c) **E100-TiO_2-10%**. d) **E100-TiO_2-50%**. In both **E100-TiO_2-x%** composites, we observe a lower number of filler particles than their respective SiO_2-filled counterparts. The surface area of the SiO_2 particles is more than two times that of the TiO_2 particles used. In addition, the filler volume fraction varies due to the higher density (ρ = 4.23) of TiO_2 particles compared to SiO_2 particles (ρ = 2.2).

Please do not adjust margins

Please do not adjust margins

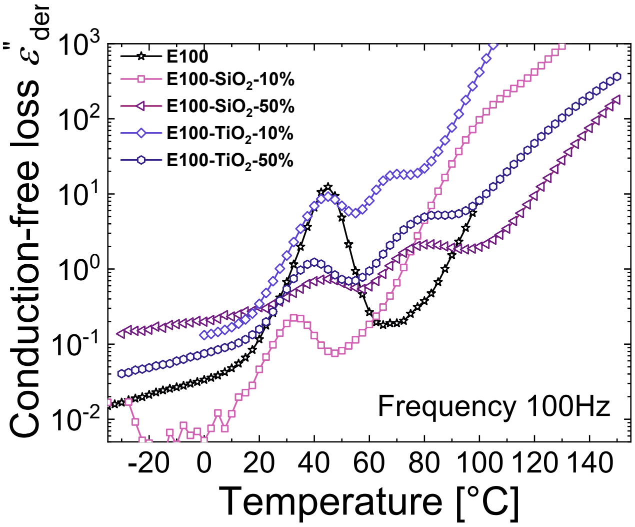

Figure S 18. ε''_{der} curves of the **E100** and **E100-MO$_2$-x%** at 100 Hz. An interfacial T_g is seen in all composites. In addition, the strength of both the relaxations can be observed. An increase in the absorbed interfacial fraction reduces the strength of the second glass-transition relaxation in 50 wt.% loaded SiO_2 and TiO_2 composites. This observation also supports the decrease in the $T_{g,int}$ observed in these samples. Hence, the **E100-TiO$_2$-50%** with a smaller surface area, despite its stronger interaction with the matrix (compared to SiO_2) still shows a similar relaxation strength as the **E100-SiO$_2$-50%**.

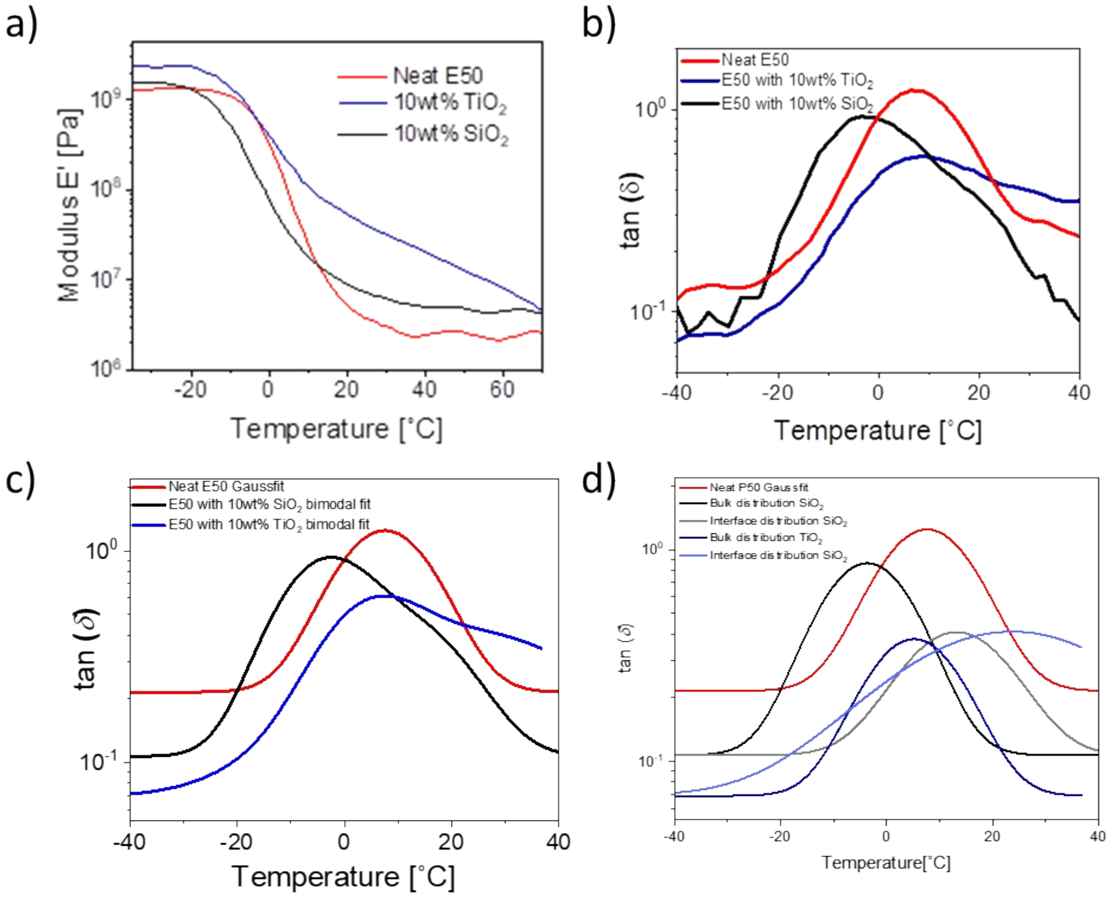

Figure S19. Temperature DMA of **E50**, **E50-TiO$_2$-10** wt% and **E50-SiO$_2$-10** wt%.a) E'-Modulus of the three samples between -40 °C up to +70 °C b) tan(δ) of the three samples between -40 °C up to +40 °C. c) Gaussian and bimodal fit of the tan(δ). d) Fitted distributions of the two individual processes derived from the bimodal fit.

This journal is © The Royal Society of Chemistry 20xx

Please do not adjust margins

Please do not adjust margins

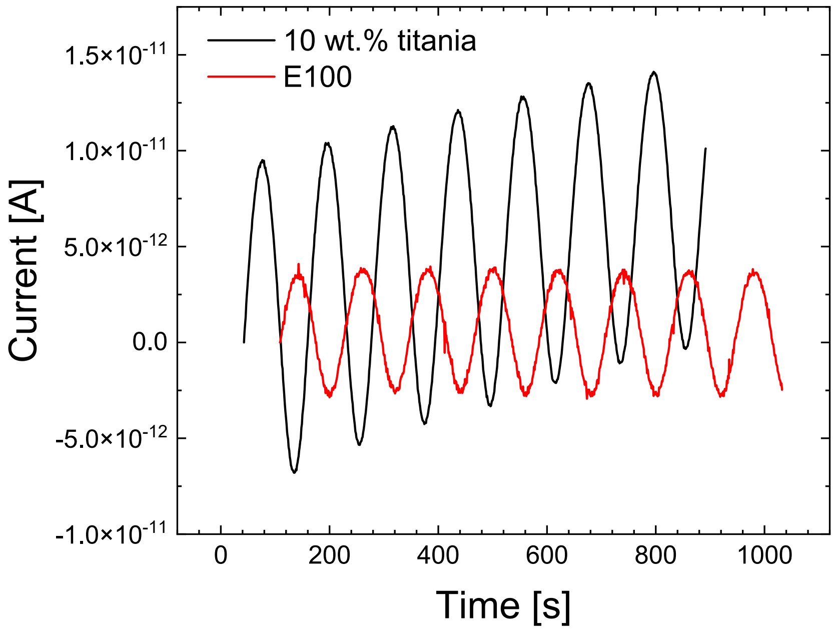

Figure S20. Quasi-static pyroelectric current measured in a 10 wt% TiO_2 (E_p = 2.5 V/µm) in comparison to that from neat **E100** polymer (E_p = 5 V/µm) at 25 °C.

Please do not adjust margins

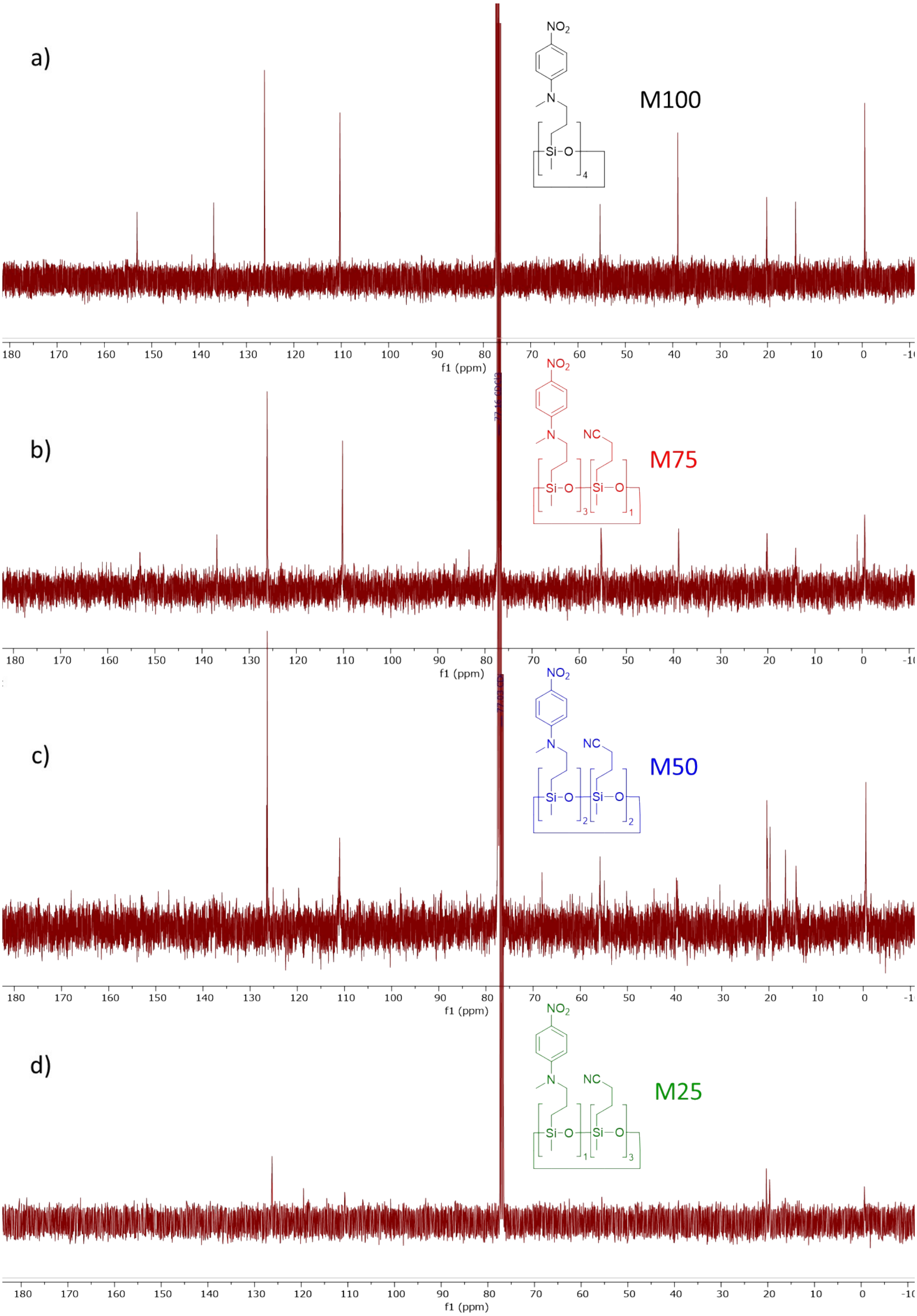

Figure S 21. ^{13}C NMR in $CDCl_3$ of the monomers: a) **M100**, b) **M75**, c) **M50**, and d) **M25**.

This journal is © The Royal Society of Chemistry 20xx

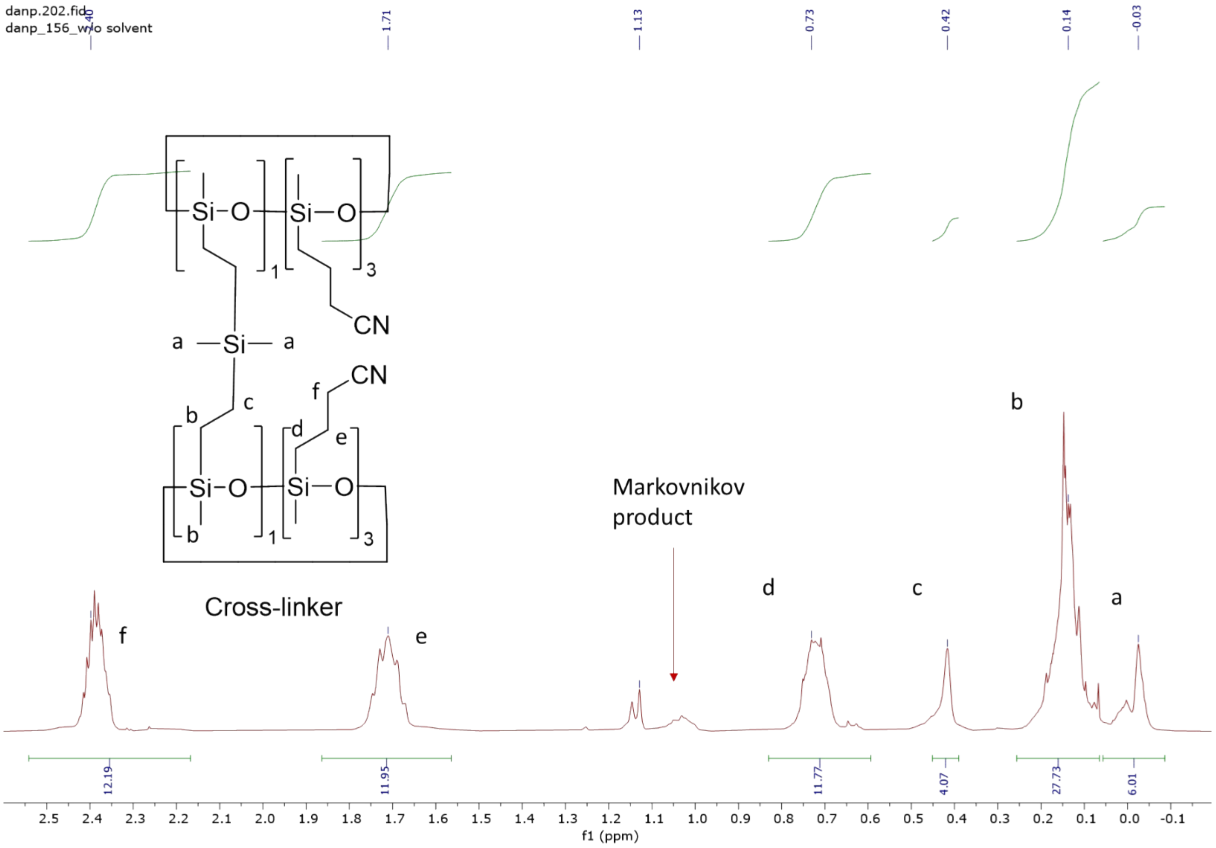

Figure S22. ^1H NMR of the cross-linker in $CDCl_3$.

Please do not adjust margins

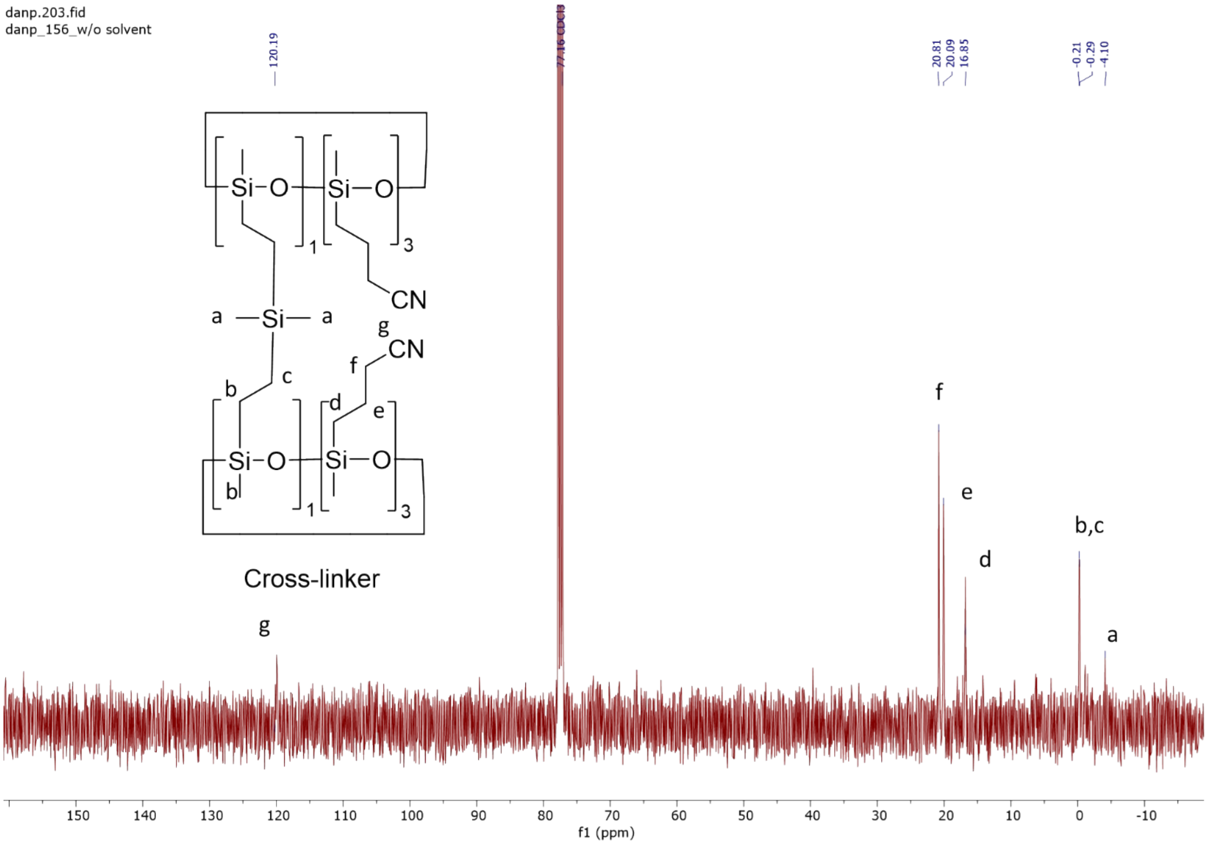

Figure S23. ^{13}C NMR of the cross-linker in $CDCl_3$.

 This journal is © The Royal Society of Chemistry 20xx

Please do not adjust margins
